# Structure-Based Analysis Reveals Cancer Missense Mutations Target Protein Interaction Interfaces

**DOI:** 10.1371/journal.pone.0152929

**Published:** 2016-04-04

**Authors:** H. Billur Engin, Jason F. Kreisberg, Hannah Carter

**Affiliations:** Division of Medical Genetics, Department of Medicine, University of California, San Diego, 9500 Gilman Dr., La Jolla, CA, 92093, United States of America; Indian Institute of Science, INDIA

## Abstract

Recently it has been shown that cancer mutations selectively target protein-protein interactions. We hypothesized that mutations affecting distinct protein interactions involving established cancer genes could contribute to tumor heterogeneity, and that novel mechanistic insights might be gained into tumorigenesis by investigating protein interactions under positive selection in cancer. To identify protein interactions under positive selection in cancer, we mapped over 1.2 million nonsynonymous somatic cancer mutations onto 4,896 experimentally determined protein structures and analyzed their spatial distribution. In total, 20% of mutations on the surface of known cancer genes perturbed protein-protein interactions (PPIs), and this enrichment for PPI interfaces was observed for both tumor suppressors (Odds Ratio 1.28, P-value < 10^−4^) and oncogenes (Odds Ratio 1.17, P-value < 10^−3^). To study this further, we constructed a bipartite network representing structurally resolved PPIs from all available human complexes in the Protein Data Bank (2,864 proteins, 3,072 PPIs). Analysis of frequently mutated cancer genes within this network revealed that tumor-suppressors, but not oncogenes, are significantly enriched with functional mutations in homo-oligomerization regions (Odds Ratio 3.68, P-Value < 10^−8^). We present two important examples, TP53 and beta-2-microglobulin, for which the patterns of somatic mutations at interfaces provide insights into specifically perturbed biological circuits. In patients with TP53 mutations, patient survival correlated with the specific interactions that were perturbed. Moreover, we investigated mutations at the interface of protein-nucleotide interactions and observed an unexpected number of missense mutations but not silent mutations occurring within DNA and RNA binding sites. Finally, we provide a resource of 3,072 PPI interfaces ranked according to their mutation rates. Analysis of this list highlights 282 novel candidate cancer genes that encode proteins participating in interactions that are perturbed recurrently across tumors. In summary, mutation of specific protein interactions is an important contributor to tumor heterogeneity and may have important implications for clinical outcomes.

## Introduction

Tumor genome sequencing is increasingly being used to inform clinical decisions for cancer patients. This is motivated in large part by the availability of targeted therapies that selectively kill cells harboring specific protein-coding mutations. However, each tumor is characterized by a unique profile of mutated genes with little overlap between patients. Few of these genes can be effectively targeted and nearly one fourth of patients do not harbor any clinically actionable mutations[1]. The recent discovery that protein-protein interfaces are enriched with cancer mutations suggests that interaction-specific perturbations may play a critical role in tumorigenesis[2]. Thus investigation of patterns of mutation at protein interaction interfaces in tumors may provide novel insights into mechanism of tumorigenesis and into factors that influence patient outcome and response to therapy.

Most tumor genome analyses to date assign a binary functional status to a gene or pathway if it harbors a mutation predicted to alter protein activity; however, this simple classification may be inadequate. Recent studies of Mendelian diseases, a class of genetic disorders that include cancer predisposing syndromes, have found that distinct mutations in the same gene can cause different phenotypes. In 2009, Zhong *et al*.[3] suggested that mutations that completely disrupt a protein’s activity have different functional consequences than mutations that affect a subset of protein-protein interaction (PPI). This idea of “edgetic” perturbations in diseases motivated several groups to integrate structural bioinformatics with biological networks to construct structurally resolved PPI networks[4, 5]. Using these networks, three independent groups observed that many Mendelian disease mutations are located at interaction interfaces[5–7]. Notably, mutations affecting different interfaces on the same protein were sometimes associated with distinct disease phenotypes, whereas mutations on interacting partners more frequently caused the same phenotype[5, 8]. More recently Sahni *et al*.[9] experimentally confirmed that mutations affecting distinct protein-protein and protein-DNA interactions cause different molecular phenotypes.

Somatic mutations arising in tumors have characteristics similar to Mendelian disease mutations[10], and thus may also cause interface-specific molecular phenotypes. During tumorigenesis, cancer genes—oncogenes and tumor suppressors—are frequently inappropriately activated or inactivated by mutations respectively. Protein sequence changes are more likely to inactivate a protein than to activate it[11]. Indeed, oncogenes are characterized by frequently mutated hotspots (*e*.*g*., PIK3CA residues E542, E545 and H1047 or KRAS residues G12, G13 and Q61) whereas tumor suppressors tend to display a random distribution of protein altering mutations[12]. However, tumor suppressors have also been shown to harbor non-random patterns of somatic mutation at the level of protein domains[13, 14]. Miller *et al*. recently grouped mutations from The Cancer Genome Atlas (TCGA) by gene families with shared homologous domains to identify rare functional mutations similarly perturbing a domain [15]. More broadly, cancer genes have different somatic mutation rates in functionally important regions [16], harbor an excess of mutations at protein interaction interfaces [2], and spatial localization of a mutation is correlated with oncogenicity[17].

To aid researchers in exploring mutation distribution on their protein of interest, Vazquez *et al*.[18] recently mapped over 170,000 cancer specific single nucleotide variants (SNVs) onto Interactome3D. Several prior studies demonstrated the utility of this approach for analyzing cancer mutations; both analyses using 3D protein structure^4^ and early efforts using structurally resolved PPIs [19, 20] provided clear evidence that mutations altering PPIs are important for phenotypic outcomes. We recently reported a strategy to extract more specific cancer pathways from a PPI network by using edge-specific cancer mutations profiles[21]. Forty-three cancer genes were found to harbor mutations at core residues and/or at distinct protein interfaces, and thus could potentially contribute to multiple molecular phenotypes. However, the extent to which cancer mutations perturb protein interaction networks and how these perturbations contribute to phenotypic diversity remains largely unexplored.

**Fig 1:**
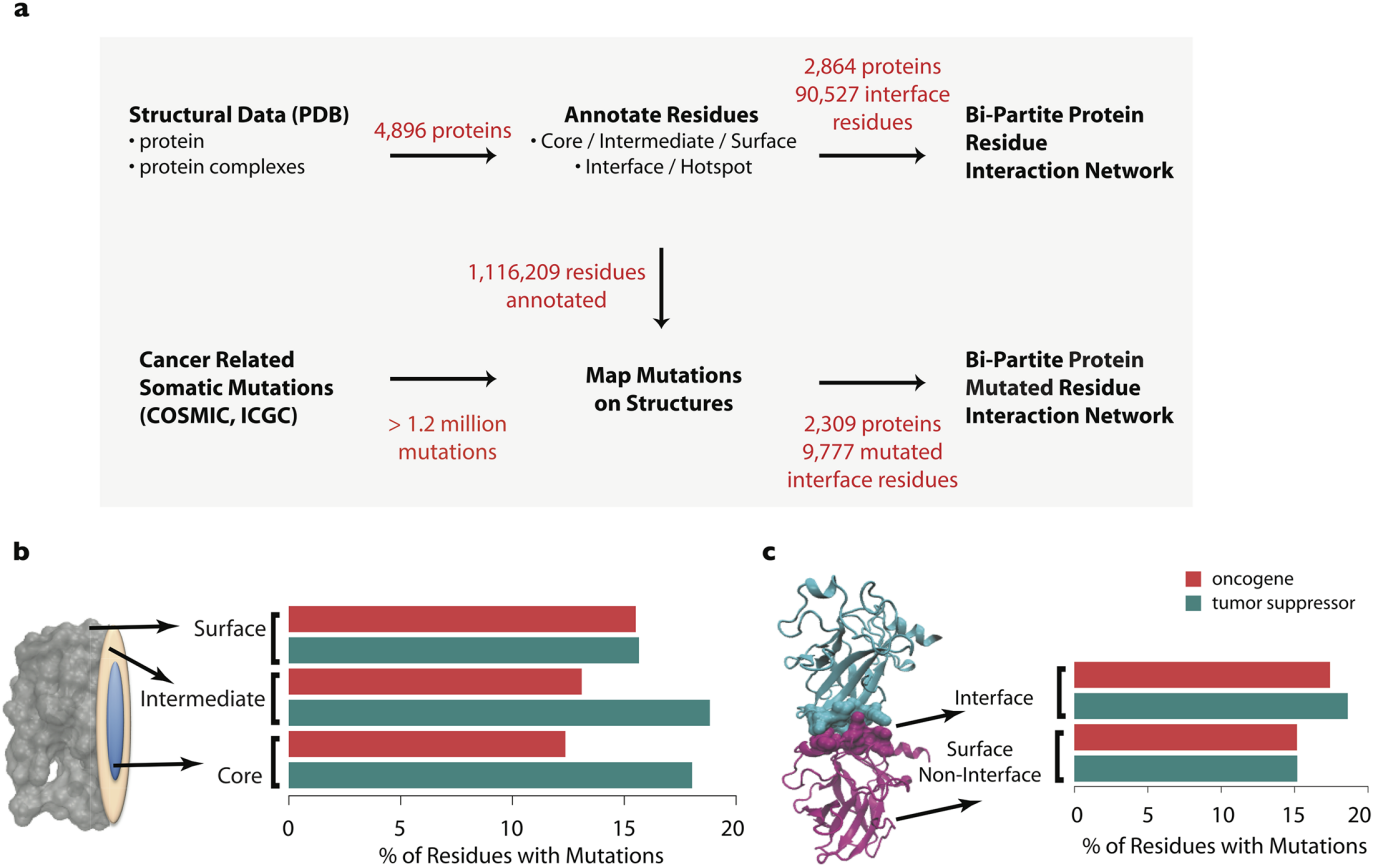
An overview of the analyses performed. **a)** A workflow describing the data processing steps from protein structures in the PDB and cancer-related somatic mutations in COSMIC and ICGC to residue-level bi-partite protein interaction networks. **b)** The percentage of residues within surface, intermediate and core regions that harbor mutations for oncogenes (n = 56) and tumor suppressors (n = 47) with 3D structures. **c)** Focusing only on surface residues, the percentage of residues within interface and non-interface regions that harbor mutations for oncogenes and tumor suppressors with 3D structures.

To investigate the mechanisms by which cancer mutations peturb protein-protein interactions, we analyzed the distribution of 1,297,414 somatic missense mutations using 3D protein structures. We first focused on a set of 103 genes that are frequently mutated in cancer due to strong positive selection in tumors. These genes are likely to be enriched for causal mutations. We then extended our analysis to interaction partners and finally to all genes for which protein structures were available. To explore whether somatic mutations contributed to tumorigenesis by perturbing protein interactions, we built a PPI network incorporating atomic level details of interfaces, which also included protein-DNA and protein-RNA interactions (Fig 1a). We found that specific molecular interactions are targeted during tumorigenesis for many known cancer genes and that mutations affecting different interfaces on the same protein can be associated with distinct patient outcomes. These findings are consistent with those of Porta-Pardo *et al*. [2], who recently catalogued cancer driver interactions based on mutations from a smaller cancer cohort using a hybrid structural proteomics dataset consisting of experimental and modeled protein complexes.

## Results

### Large-scale Analysis of Missense Mutations Affecting Cancer Genes

We performed an in depth analysis of the localization of cancer-associated missense mutations on three-dimensional protein structure, considering only somatic mutations which are present in the tumor exome but not in patient-matched normal tissue. A total of 1,297,414 somatic mutations observed in 17,028 tumor exomes from the combined International Cancer Genome Consortium (ICGC)[22] and Catalogue of Somatic Mutations In Cancer (COSMIC)[23] databases were mapped onto human protein structures from the protein databank (PDB)[24] (Fig 1a) where available. Each amino acid position in every protein structure was labeled as core, intermediate or surface based on solvent accessibility. Using co-crystal structures of protein interactions, we further determined the interface residues on each protein responsible for mediating the physical interaction between binding partners. We observe a similar proportion of mutations (16%) mapping to residues on the surface of oncogenes and tumor suppressors, while oncogenes tend to have fewer mutations at intermediate (13% vs 19%) and core residues (12% vs 18%) (Fig 1b). Interestingly, tumor suppressors harbor slightly more (17% vs 19%) mutations at interface sites than oncogenes (Fig 1c).

### Missense Mutations in Cancer Genes are More Frequent at Core and Interface Residues

Our study focused initially on 138 genes known to play a causal role in cancer[12] (S1a–S1c Fig). Of the 138 cancer genes, 103 (56 tumor-suppressors, 47 oncogenes) had monomeric structural information, and 89 had one or more co-crystal structures in complex with a binding partner. We compared results for cancer genes to another 4600 human proteins for which we had structural information. Since these genes are most likely predominantly not cancer-related, we expect they are representative of a population not undergoing strong selection for causal cancer mutations.

**Fig 2:**
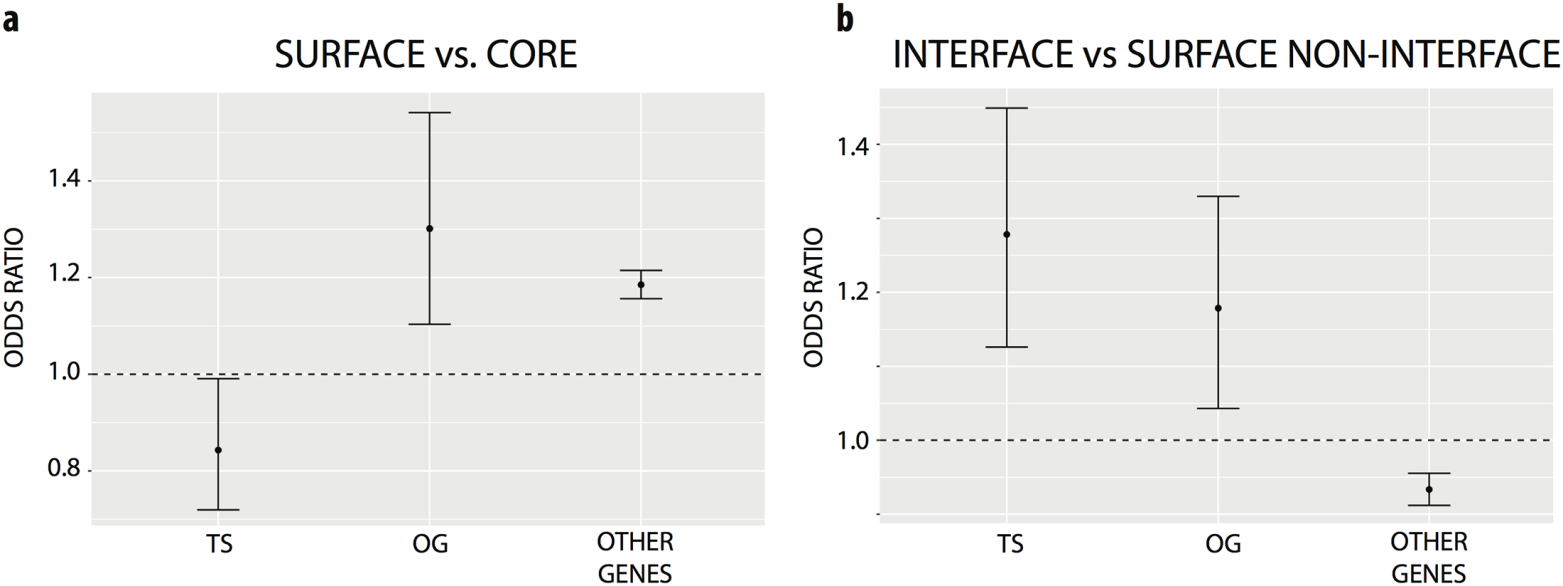
Characterizing the structural location of missense mutations in tumor suppressors (TS), oncogenes (OG) and other genes. Fisher’s exact tests were performed separately for each set of genes. Shown are the odds ratios and 95% confidence intervals within each set of genes when making comparing the number of mutations located at **a)** surface versus core residues, **b)** surface interface versus surface non-interface residues.

Two-sided Fisher’s exact tests were used to test whether the incidence of mutated residues in a particular structural niche (core, intermediate, surface or interface) deviated from random expectation for proteins encoding oncogenes, tumor suppressors or other genes. We observed that mutated residues tended to occur in the core of tumor suppressors (P-value < 3.6 x 10^−2^, Odds Ratio 1.19) but on the surface of oncogenes (P-value < 1.3x10^-6^, Odds Ratio 1.30) and other genes (P-value < 2.2×10^−16^, Odds Ratio 1.18) (Fig 2a and S1 Table). As core mutations are often destabilizing to a protein’s 3D structure[25], this finding is consistent with tumor suppressor genes harboring frequent loss-of function mutations. Analyzing the frequency of specific mutations across tumors, we observed that the most recurrent mutations in tumor suppressors occurred primarily at core residues (S1d Fig), whereas recurrent mutations in oncogenes occurred primarily at interface residues (S1e Fig).

We next focused on surface residues, dividing them into residues at protein interaction interfaces versus other surface residues. In total 20% (783) of the 3837 genetic variants mapped to surface residues on cancer gene products occurred at PPI interfaces. Analyzing each group of genes separately, we observed an excess of missense mutations at interface residues relative to surface non-interface residues in both tumor suppressors (P-value < 1.4x10^-4^, Odds Ratio 1.28) and oncogenes (P-value < 7.92x10^-3^, Odds Ratio 1.17) (Fig 2b and S1 Table), but not other genes. Since different interfaces mediate distinct protein activities, this finding suggests that specific activities of both tumor suppressors and oncogenes are targeted in cancer, and complete loss of function may not be necessary for some tumor suppressor genes to promote tumorigenesis.

Silent mutations are often used to represent the background mutation rate in tumorigenesis [26–28], since most silent mutations are unlikely to alter protein activity and therefore are probably not undergoing positive selection. We repeated our tests using silent mutations to determine whether random mutations showed similar preference for core, surface and interface residues. As we did not observe the same trends (S2 Fig), this suggests that positive selection is acting to specifically target protein sequence alterations to functionally important sites on proteins in cancer.

### Core and Interface Regions are More Likely to Harbor Functional Mutations

We annotated all somatic missense mutations with functional scores generated by VEST[29]. While not cancer-specific, VEST scores can still be helpful for determining whether the observed mutations are likely to perturb protein activity (Methods). We observed that mutations in the core of proteins and those affecting interface residues were more likely to receive functional VEST scores, while mutations at surface non-interface residues had a bimodal distribution, suggesting that these mutations may be affecting as yet undiscovered binding sites or other functionally important classes of residue on the surface (S1f Fig). In general, functional mutations were enriched at interface residues compared with surface non-interface residues (P-value < 2.6×10^−2^, Odds Ratio 1.06)(S2 Table), which could indicate that amino acid substitutions at an interface are more likely to have functional consequences in general, or that functional mutations at interfaces are under positive selection in cancer, even amongst genes that are not frequently mutated.

Functional mutations at protein interaction interfaces could affect protein binding affinities. Nishi *et al*. [20] found that 97 missense mutations from 68 genes in general had a destabilizing effect on binding affinities of protein-protein interactions. To investigate this on a larger scale, we calculated the change in binding free energy between wild type and mutant cancer protein sequences for 5857 interface amino acid substitutions reported in ICGC and COSMIC (for this analysis we used all mutations in COSMIC). Of these, 1225 altered binding affinity (S1 File): 903 were predicted to destabilize interactions, whereas the other 322 were predicted to stabilize interactions. To determine whether the effect on binding affinity was consistent with the function of the interactions we annotated them as activating or inhibitory using the Reactome Pathway Database[30]. One hundred fifty two interactions could be annotated as activating and 15 as inhibitory. We observed that activating interfaces on tumor suppressor genes were highly enriched for destabilizing mutations as compared with activating interfaces on oncogenes (Fisher’s Exact Test P-value < 8.36×10^−4^, Odds Ratio 3.65) (S3 and S4 Tables).

### A Bipartite Protein-Residue Interaction Network Highlights Mutation Patterns in Cancer Genes

**Fig 3:**
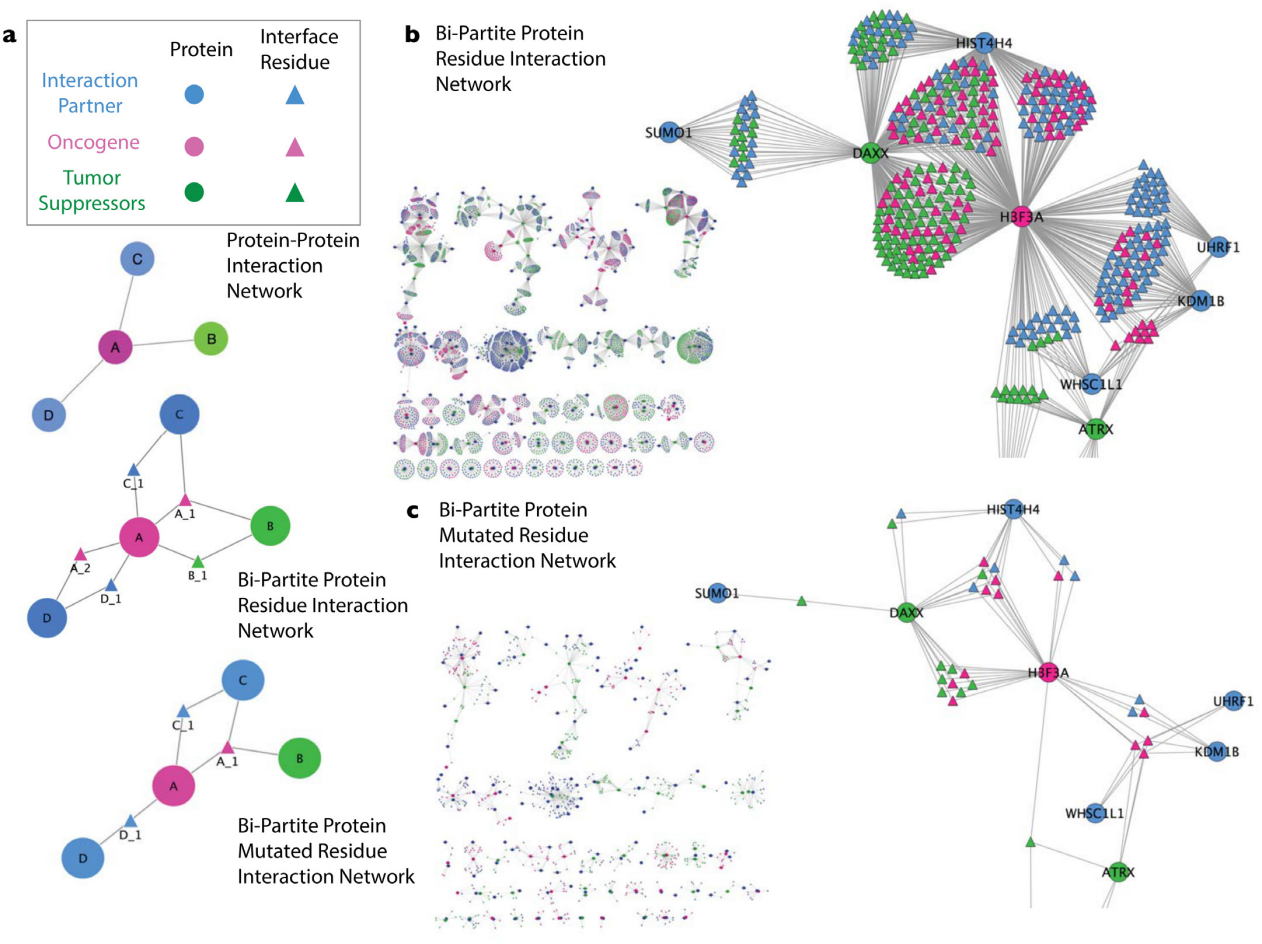
A bipartite protein-residue interaction network constructed for cancer genes and their interaction partners. Edges involved in self-interaction are not shown. **a)** An example of a network describing how proteins, interface residues and mutations are represented in the bipartite network model. In a protein-protein interaction network, the nodes representing proteins A–D are directly connected to one another. In our bi-partite protein residue interaction network, interface residues are displayed between proteins. For example, residue 1 on protein A (A_1) is involved in the protein-protein interface between A and B along with A and C. Residues mutated in cancer are shown in the bi-partite protein mutated residue interaction network. For example, A_1 is mutated in at least one cancer in one patient whereas residue B_1 –present above but absent here—is not. **b)** A bipartite network showing cancer genes and their immediate interactors. **c)** A bipartite network displaying only residues that were mutated in one or more tumors. The circled interface residues interact with multiple proteins.

To investigate the extent to which cancer mutations at interface residues target specific protein interaction interfaces, we constructed a bi-partite network of protein-interactions. This network explicitly depicts the residues mediating PPIs on each partner, thus the network includes two distinct classes of nodes: circles represent proteins and triangles represent amino acid residues (Fig 3a). We focused first on the subnetwork of frequently altered cancer genes and their interaction partners (Fig 3), This subnetwork consisted of 185 protein nodes of which 65 are cancer genes and 120 are interaction partners. Residues mediating homo-dimerization were not included in this figure but are present in the complete network. In Fig 3b, all residues participating in interfaces are shown, whereas Fig 3c shows only the interface residues that are mutated in cancer.

We found that on average, 2.2 binding sites per cancer gene harbored mutations, with some tumor suppressors (FUBP1, KMT2D, NOTCH2 and MLH1) and oncogenes (CCND1 and SKP2) having no interface mutations and some cancer genes having mutations at multiple distinct interfaces (including TP53 with mutations at 8 different interfaces, CTNNB1 with mutations at 7 different interafaces, APC with mutations at 6 different interfaces and EGFR with mutations at 4 different interfaces). This observation suggests that phenotypic pleiotropy arising from distinct alteration of interaction profiles of cancer genes could be contribute to tumor heterogeneity.

Two particularly interesting regions of the bi-partite network center on tumor suppressor genes B2M and TP53. These network modules show distinct patterns of mutation localization at interfaces that are suggestive of different selective pressures acting to target mutations in each case. We describe these two modules in more detail in the following sections.

### B2M Network Module

**Fig 4:**
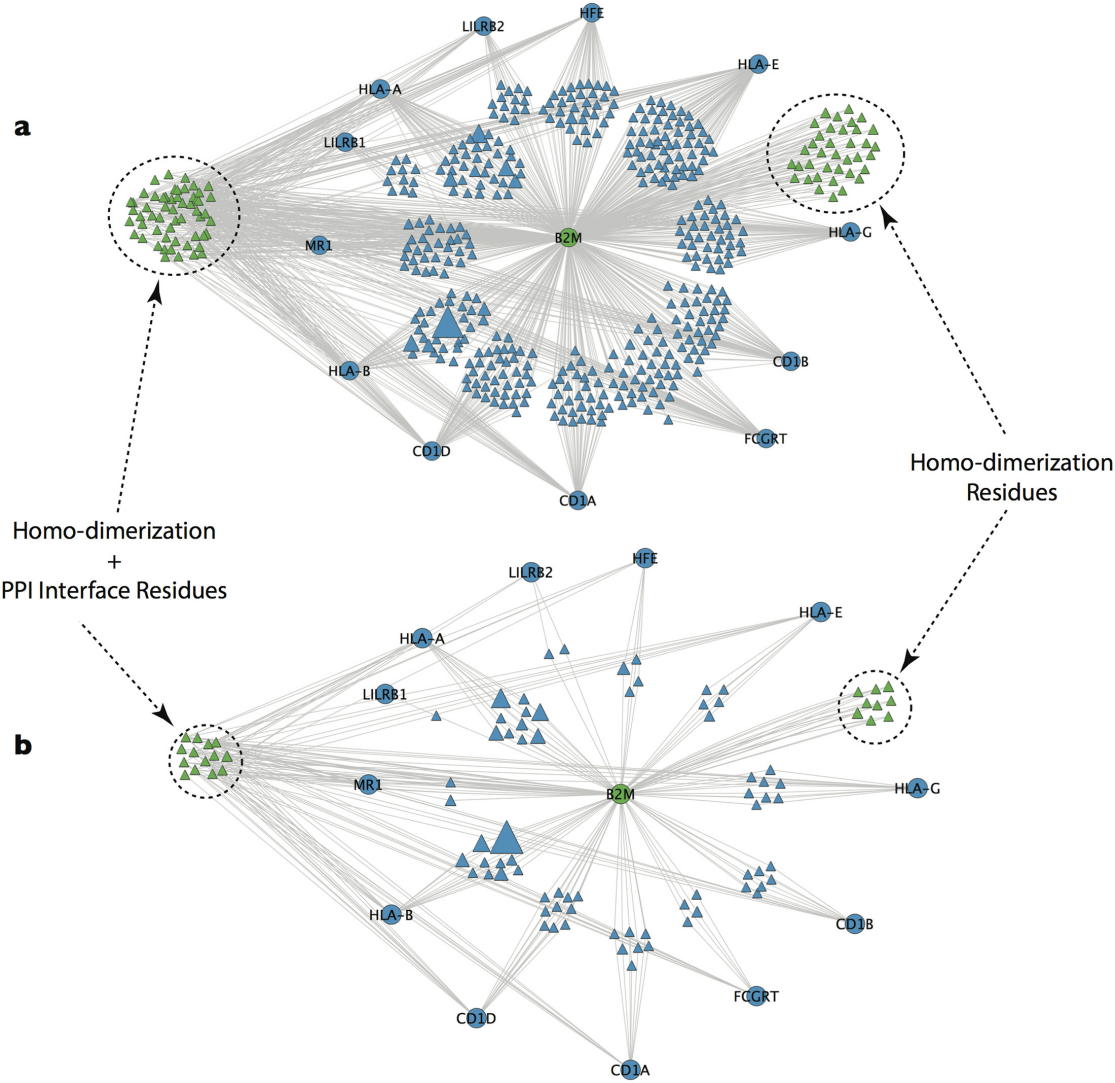
Bipartite B2M interaction networks. **a)** A bipartite network of the tumor suppressor B2M, its partners and the interface residues by which they interact. **b)** A bipartite network showing only the subset of residues that were observed to harbor missense mutations in cancer patients. The size here of the residue nodes represent the number of tumors in which the residue was mutated.

Unlike most cancer genes in our network, interface mutations affecting the tumor suppressor beta-2 microglobulin (B2M) were located predominantly on partner genes. Most of those partners compete with each other to bind the same site on B2M (Fig 4a). The most frequently observed mutations among B2M partners were residues 121 and 33 on HLA-A and residues 140 and 118 on HLA-B.

Apart from LILRB1 and LILRB2, the other 10 interaction partners of B2M (Fig 4b) are all involved with antigen presentation. These include MHC class 1 proteins (HLA-A, HLA-B, HLA-G, HLA-E) and members of a class of closely related proteins involved with the presentation of non-peptide antigens (CD1A, CD1B, CD1D, HFE, MR1 and FCGRT). Surface expression and antigen presentation of MHC class I requires binding to B2M[31]. MHC class 1 genes are highly polymorphic, which enables them to present a variety of different endogenous peptides[31]. Mutations affecting B2M binding with partners in the antigen presentation pathway could decrease the efficiency of self-antigen presentation and thereby facilitate immune response evasion by tumor cells. The enrichment of mutations at partner interfaces may reflect that selection in tumors is acting to interfere specifically with the presentation of self-antigens, such that tumors with different mutation profiles need to interfere with different aspects of the antigen presentation pathway.

### TP53 Network Module

TP53 is the most commonly mutated gene in human cancers with mutations distributed throughout the open reading frame^3^. Mutations in this gene have been reported to have different consequences for TP53 activity[32]; some of the mutations cause gain of function while others suppress TP53. Even distinct amino acid substitutions at the same residue can lead to different phenotypes[32]. Here we used the protein-residue bipartite interaction network of TP53 to examine the possible biological outcomes of distinct mutations.

**Fig 5:**
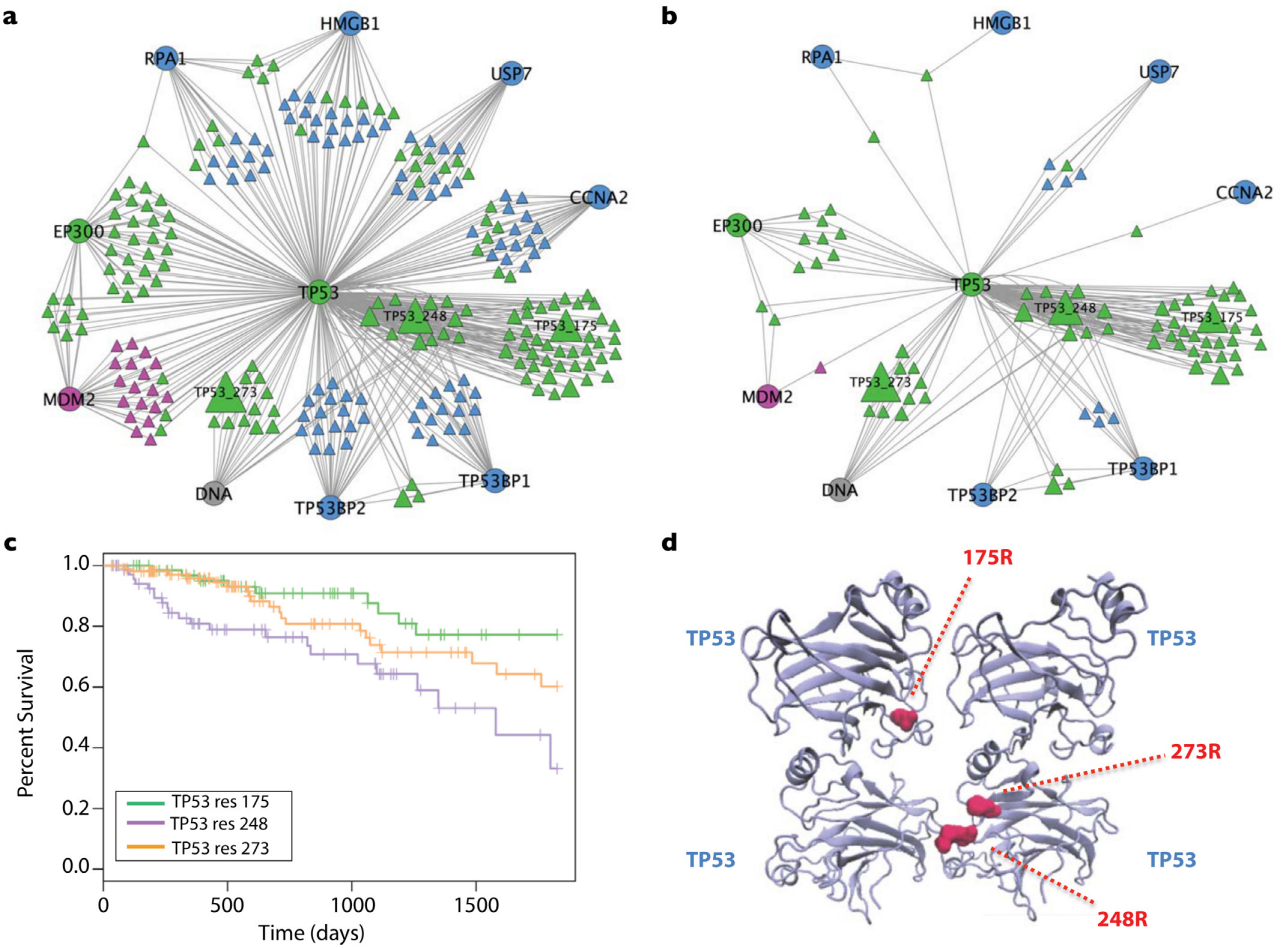
Mutations affecting TP53 interaction interfaces and their impact on patient survival. **a)** A bipartite network of the tumor suppressor TP53, its partners and the interface residues by which they interact. **b)** A bipartite network showing only the subset of residues observed to harbor missense mutations in cancer patients. The size here of the residue nodes represent the number of tumors in which the residue was mutated. **c)** A Kaplan Meier survival plot of patients from TCGA harboring mutations in TP53 at residues 175, 248 or 273. **d)** The TP53 mutations R175, R273 and R248 displayed on the crystal structure of TP53 as a homotetramer.

Several of the mutated interface residues in the TP53 network module mediate multiple protein interactions. Mutated TP53 residues 18 and 27 interact with both MDM2 and EP300 (Fig 5a and 5b). Shared residues are particularly interesting because mutations at these sites could simultaneously interfere with multiple intracellular signals, or could shift the equilibrium of binding between interaction partners. According to Kohn’s 2-state model[33] EP300 has two roles in the TP53 network. When in the inactive state (in the absence of cellular stress), TP53 can be inactivated via ubiquitination by either MDM2 or EP300. In this scenario, EP300 cooperates with MDM2. However in the active state (triggered by DNA damage) phosphorylation of TP53 (residues 18 or 20) inhibits MDM2 binding, while it promotes EP300 binding. MDM2 is a negative regulator of TP53 whereas EP300 stimulates TP53’s transcriptional activity. Thus EP300 has an opposing role to MDM2 in the TP53 active state. By destabilizing the TP53-EP300 interaction, which is unfavorable for tumor progression, mutations at these residues could specifically inhibit EP300 binding, thereby freeing the binding site to interact with MDM2.

TP53 residues 181, 247 and 249 interact with TP53BP1 and TP53BP2 (Fig 5a and 5b). TP53BP1 contributes to DNA repair and cell cycle control, as well as enhancing TP53-mediated transcriptional activity[34], and TP53BP2 enhances damage-induced apoptosis[35]. In contrast to the MDM2-EP300 example, the most advantageous outcome for the tumor would seem to result if mutations at residues 181, 247 and 249 compromised both TP53BP1 and TP53BP2 interactions. Interestingly, mutations at residue 249 were predicted to stabilize the interaction with TP53BP1 but destabilize the interaction with TP53BP2 (S1 File) suggesting a more complex role for these TP53 binding partners in tumorigenesis

TP53 residue 45 is important for binding to RPA1 and HGMB1. The TP53-RPA1 complex is important for homologous recombination and essential for tumor suppression[36], whereas HMGB1 has both oncogenic and tumorigenic activities[37]. It has been proposed that in the absence of TP53, HMBG1 promotes autophagy and HMGB1-mediated autophagy stimulates tumor cell survival through TP53-dependent processes[38]. Disrupting both RPA1 and HMBG1 interactions with TP53 may therefore be advantageous for tumor maintenance.

Certain mutations in TP53 are known to have a prognostic value for cancer patients. These mutations are categorized according to whether they affect DNA binding capacity (R248Q, R273H) or overall protein stability (R249S, G245S, R175H and R282W)[39]. Poeta *et al*.[40] classified TP53 mutations as disruptive or non-disruptive according to whether or not they are located in the DNA binding domain (DBD). Mutations in the disruptive category were associated with shorter survival. Particular mutation hotspots (248, 273 and 175) were recently observed to influence chemotherapy sensitivity and overall survival in ovarian cancer[41]. Although the DNA binding and oligomerization domains of TP53 are usually treated separately, our interface mappings highlight that many amino acids involved in TP53 dimerization are within the DBD, a fact that has been reported previously[42].

While both residues 248 and 273 contribute to DNA binding, their topological affects on the PPI network differ. We observed 3 mutational hotspots (frequently mutated residues) that are involved with different sets of interactions: TP53 residue 273 specifically affects DNA binding, residue 175 specifically affects TP53 oligomerization and residue 248 is important for oligomerization, binding to DNA and interactions with two protein partners, TP53BP1 and TP53BP2. When patients were grouped according to mutation at these three sites, we observed a statistically significant difference in survival trends (Chi-Square = 11.1, P-value < 3.8×10^−3^, Log-Rank Test) (Fig 5c). The three-dimensional localization of residues 175, 248 and 273 on a TP53 tetramer is shown in Fig 5d.

### Functional Mutations Are Enriched at Tumor Suppressor but not Oncogene Homo-Oligomerization Sites

**Fig 6:**
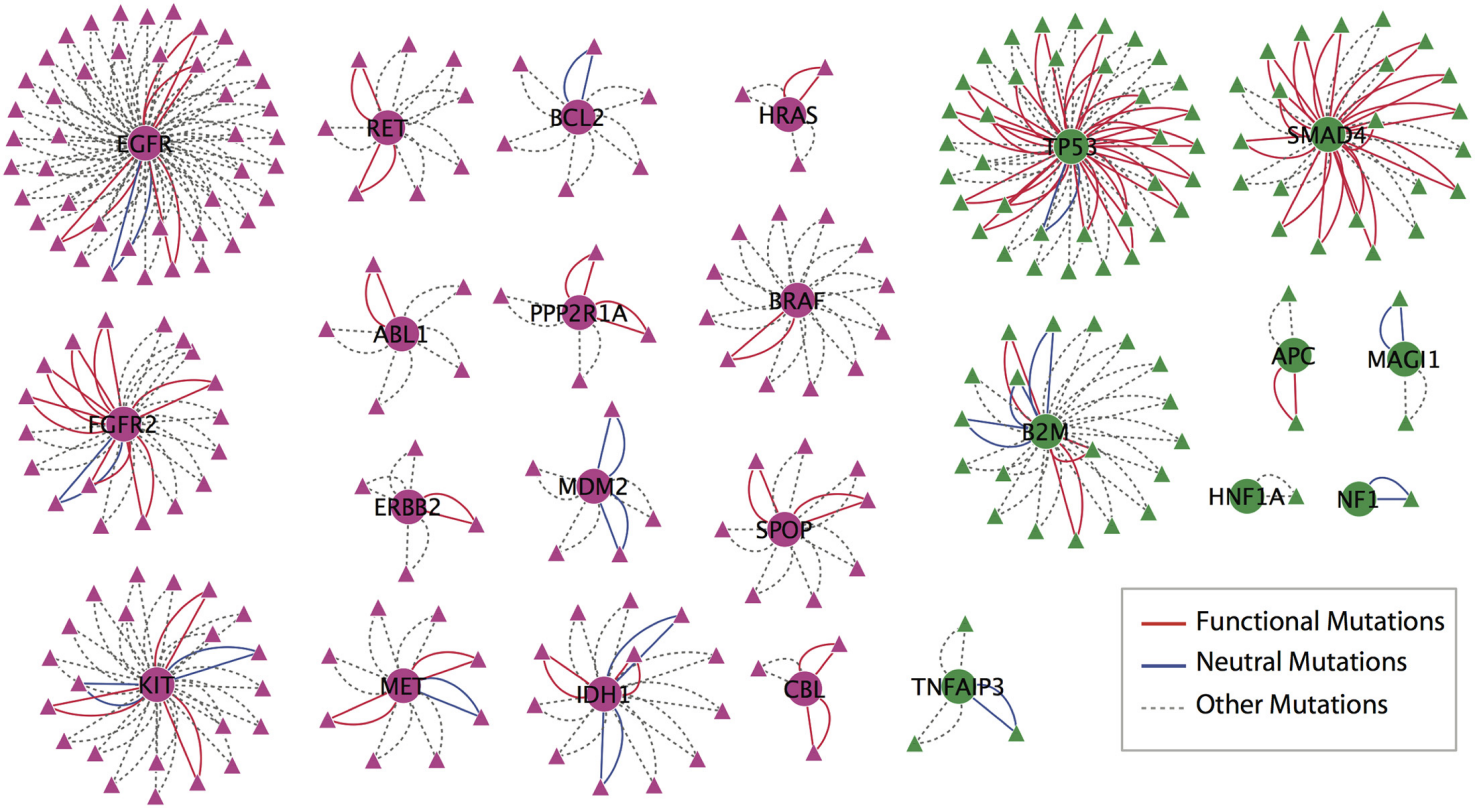
Bipartite protein-residue interaction networks of 34 cancer genes known to form homo-oligomers displaying only those residues involved in oligomerization. Edges are colored according to functional predictions made using VEST. Red lines indicate that mutations affecting that residue were predicted to be functional (VEST > 0.75), blue lines indicate a neutral prediction (VEST < 0.25), and dashed grey lines indicate mutations could not confidently be assigned a functional or neutral label.

Mutations could be mapped onto homo-oligomerization sites of 46 cancer genes. Since crystallization sometimes detects protein-protein contacts that do not happen in the cell, our analysis focused on 23 of the cancer genes that were confirmed to form biological oligomerizations by PISA[43], and directly from the literature (S5 and S6 Tables). Since failure to oligomerize is likely to interfere with protein function, we found it interesting that mutated oligomerization sites were approximately equally represented among oncogenes and tumor suppressors (Fig 6). We speculated that mutations at these sites in tumor suppressors but not oncogenes would be enriched for functional amino acid substitutions. To test this, VEST score distributions for mutations were compared across oligomerization sites in oncogenes, tumor suppressors and other genes. We found that tumor suppressors are significantly enriched with functional mutations in their homo-oligomerization regions compared to other genes (P-value < 1.73×10^−8^, Odds Ratio 3.68) (S7 Table) but did not observe this enrichment for oncogenes (Fig 6).

### Nucleic Acid Binding Sites Harbor an Unexpected Number of Missense Mutations

We next examined protein-DNA binding sites in our network. Mendelian mutations at DNA binding sites were experimentally found either to abrogate DNA binding or to alter DNA binding specificity for binding motifs in DNA sequence[9]. The PDB included structures for 10 tumor suppressors, 2 oncogenes and 168 additional genes bound to DNA (S8 Table). Among these, 9 tumor suppressors, 1 oncogene and 131 other genes had mutations in their DNA binding regions (S9 Table). DNA binding regions generally do not overlap with protein-interacting regions of proteins in Mendelian diseases[9]. The proteins in our network similarly showed almost no overlap of protein interaction sites with structurally resolved DNA binding interfaces (P-value < 8.86×10^−5^, Odds Ratio 0.77). In addition, an unexpected number of missense mutations but not silent mutations occurred within DNA binding sites (P-value < 3.38×10^−3^, Odds Ratio 1.19) (S10 Table), suggesting that the DNA binding activity of some of these proteins could be important for tumorigenesis.

Of the 180 DNA-binding proteins in our network, ten were transcriptional master regulators (ETS1, SRF, FOXO4, GATA3, HNF1B, HNF4A, MAX, MYC, NFKB1 and NFATC1[44, 45]), eight of which (SRF and HNF1B are the exceptions) had mutations in their DNA binding regions. Transcriptional master regulators, genes at the top of the regulatory hierarchy, are capable of controlling the expression of multiple target genes and are determinants of cell fate. Thus mutations in these genes that either abrogate DNA binding or alter its specificity are likely to cause widespread changes to gene expression.

We hypothesized that mutations altering RNA binding sites of proteins would have similar consequences to those altering DNA binding sites. Our network included one RNA-binding oncogene and 73 RNA-binding other genes supported by co-crystal structures in the PDB (S11 Table). Unlike what has been reported for DNA binding domains, we did not observe mutual exclusivity between residues mediating protein-protein interactions and those mediating protein-RNA interactions. Fifty-six of the genes, including the single oncogene, harbored mutations in their RNA binding region (S12 Table). This represented an overall enrichment for somatic mutations at RNA binding sites in these genes relative to random expectation (P-value < 1.27×10^−2^, Odds Ratio 1.23) (S13 Table).

### Protein Interactions are Targeted by Somatic Mutations in Cancer

Our results suggest that specific targeting of molecular interactions is widespread in tumorigenesis. To further investigate this, we assembled 3,072 high quality interfaces from our network, which included 199 interactions between 89 cancer genes and 145 immediate interaction partners. By grouping the reciprocal interface residues on two binding partners, we can test whether edges in our network are enriched for mutations. Taking this approach we found a strong enrichment for mutations on edges involving a cancer gene (P-value < 2.2×10^−16^, Odds Ratio 1.62) (S14 Table).

While we see strong evidence that specific interactions are targeted by cancer, we note that in many cases there is clear bias for mutations to occur on the cancer gene side of the interaction (P-value < 4.59x10^-9^, Odds Ratio 1.53) (S14 Table). Perhaps altering more than one activity of a cancer gene is beneficial to the tumor, and this is done more easily by mutating the cancer gene itself than mutating 2 or more of its interaction partners. We note that among the 145 1^st^ degree interaction partners of the cancer genes used for this study, 13 have recently been reported to frequently harbor mutations in tumors[46] (S15 Table). This suggests that genes mutated at an interaction interface that bind the protein product of a known cancer gene may also be candidate cancer genes.

To identify candidate ‘cancer interactions’, we ranked all 3,072 interactions in our network based on evidence for positive selection at the interfaces as quantified by the ratio of missense mutations to silent mutations, divided by the ratio of their probabilities given the amino acid composition of the interface (S2 File). Values of this ratio greater than 1 are suggestive of positive selection for non-synonymous mutations. While interactions involving cancer genes dominated the top of the list, we observed a number of other interactions in the network that harbored an excess of mutations. Specifically, among the 557 interactions with a value > 1, there were 282 interactors (586 if we include homo-oligomerization interactions) that were not in our list of cancer genes (S16 Table) but nonetheless participated in interactions showing evidence of positive selection during tumorigenesis. Furthermore, some interactions involving cancer genes received very low ranks, suggesting that these interactions might be important to preserve tumor cell viability. Among the 71 novel cancer driver genes reported by Porta-Pardo *et al*. [2] only 10 of them (GLUL, A2M, CYP2B6, CSNK1E, SLIT2, C3, SERPINB3, HLA-DRB1, SEC13 and TLR4) are present in our set of positively selected 557 interactions (only C3 and HLA-DRB1 if we exclude homodimer-oligomerization interactions).

## Discussion

Understanding the specific functional consequences of mutations is essential for uncovering the molecular mechanisms underlying disease and for effectively tailoring precision therapies. Many sequencing-based studies of cancer have focused on identifying genes, pathways or both based on the assumption that most mutations in a gene or pathway lead to similar phenotypes. However, genes often play multiple roles, each of which can be perturbed resulting in distinct phenotypic consequences. Mutations with pleiotropic phenotypic effects in the same gene have recently been established experimentally for inherited Mendelian disease mutations[9]. The observation that specific protein interactions are targeted by somatic cancer mutations suggests that distinct cancer mutations in oncogenes and tumor suppressors may also confer different phenotypes.

Our protein structure-based analysis of cancer genes found an excess of core mutations in tumor suppressors, consistent with their frequent inactivation in cancer. However, when only surface residues were considered, we observed that mutations in both oncogenes and tumor suppressors tended to be located at PPIs. Relative to mutations on other genes, mutations affecting tumor suppressors, but not oncogenes, were significantly enriched with functional mutations in homo-oligomerization regions (Odds Ratio 3.68, P-Value < 10^−8^) suggesting that interfering with tumor suppressor oligomerization may be a common mechanisms in tumorigenesis.

As further evidence that cancer mutations can affect specific protein activities, we found that mutations affecting known cancer genes, both oncogenes and tumor suppressors, are enriched at interfaces between proteins and their interaction partners and that many cancer genes had mutations at multiple distinct interfaces. This observation strongly suggests that mutated genes will contribute to tumorigenesis in different patients in different ways, depending on where the mutation is located. As support for this hypothesis, we show that hotspot mutations at distinct interaction interfaces on TP53 are associated with significant differences in patient survival.

Mutations on direct interaction partners of cancer genes occurred less frequently than mutations on cancer genes themselves, suggesting that there may be a greater biological advantage for mutating the interface on the side of the cancer gene. The most notable exception was B2M, where most mutations were distributed on the reciprocal interfaces of binding partners. This may reflect that antigen presentation, a process to which B2M is central, may need to be specifically altered according to the set of mutations already present in the tumor genome. Alternatively, it might suggest that even minor changes in the efficiency of antigen presentation, such as might be caused by a shift in B2M binding affinity for one partner relative to others, can be beneficial to tumorigenesis.

Our findings raise important considerations for the design and application of precision cancer therapies. In cases where a cancer gene itself is considered un-druggable, its binding partners could represent attractive alternatives. One such success is the use of Nutlin to target MDM2, in order to relieve inhibition of TP53. Furthermore, protein interaction interfaces can be specifically targeted by therapies. Extending our analysis to all genes with high quality structurally resolved interfaces, we found a number of genes with one or more interfaces harboring similar numbers of somatic mutations to known cancer genes. Several of these genes have not previously been implicated as cancer genes, and thus may represent novel therapeutic targets.

In addition to PPIs, protein-nucleotide interfaces also harbored mutations. A number of these proteins were also recently reported master transcriptional regulators (8 out of 140 genes). Sahni *et al*.[9] reported that more than 80% of tested Mendelian missense mutations in transcription factors altered DNA binding patterns, with a number of mutations resulting in new protein-DNA binding interactions. We also found that mutations were enriched in the RNA binding sites of RNA binding proteins. Other examples of somatic cancer mutations capable of altering specific aspects of protein activity including post-translational modification sites and enzyme substrate specificity have also been reported[47, 48].

In conclusion, we find that missense mutations target interactions involving oncogenes and tumor suppressors in different ways. Distinct perturbations to the protein interaction network, even those involving the same protein, can result in different patient outcomes. Understanding the mechanisms by which specific mutations influence tumor behavior and patient outcome will be essential in settings where massively parallel sequencing is used to optimize patient care. Current efforts are limited by the availability of protein structural information along with condition- and tissue-specific protein interaction networks. Our work highlights the importance of developing new tools and datasets to inform such efforts.

## Materials and Methods

### Assembling Structural Data on Proteins and Their Interactions

We downloaded the experimental structures associated with each human gene in the Protein Data Bank (PDB)[24], which corresponded to 46,867 PDB chains for 4896 proteins (~9.6 chains per protein on the average). We also collected 10,238 PDB (protein-protein) complexes. We based our structural analysis of protein complexes on 3,612 human protein-protein, 743 DNA-human protein and 198 RNA-human protein PDB complexes. We used VMD[49] to visualize protein 3D structures.

### Residue Annotation and PDB to Uniprot ID Mapping

We annotated every residue in each PDB structure as core, surface or intermediate according to the solvent accessible surface area (ASA) calculated with Naccess [50]. Residues with a relative ASA of 0 were annotated as core residue, while residues with a relative ASA greater than 15 were annotated as surface. Any residues between these thresholds were annotated as intermediate. When multiple PDB chains were available for the same protein, we used the consensus designation as the final label; ties were labeled as intermediate since the annotation was ambiguous.

Our methodology for defining the location of residues on protein structure (core/ surface/ intermediate) is limited by the availability and completeness of crystal structures in PDB. Incomplete structures could result in mislabeling of amino acids in the protein core as being located at solvent accessible sites. Residues with conflicting structural localizations (core/ intermediate/ surface) in different PDB structures of the same protein were labeled intermediate (Fig 1), and were excluded from all statistical analyses. Thus current limitations of crystallography may result in some biases in annotating residues to specific structural sites on a protein.

Residues involved in protein-protein interactions were determined as described in one of our earlier publications[21]. DNA and RNA binding residues were determined using the distance between 2 non-hydrogen atoms of amino acids and nucleotides (1 from the protein and the other from DNA/RNA). If the distance was less than 3.5A, we designated those residues as interface residues. Incomplete interfaces, those with fewer than 5 residues in either of the interacting chains, were discarded.

PDB residue positions were mapped onto Uniprot residue positions using PDBSWS webserver[51]. After this mapping, we had 4,896 proteins with residue annotations, of which 2,864 were involved in protein-protein interactions. There were 180 proteins involved in DNA binding and 73 proteins involved in RNA binding after the annotation and mapping steps. We identified 90,527 interface residues.

### Cancer Genes

For our main analysis, we used a list of 138 high confidence cancer genes, comprising 74 oncogenes and 64 tumor suppressors, assembled by Vogelstein *et al*.[12]. To determine whether genes in our network outside of the 138 had also previously been implicated in cancer, we used a list of genes published by Lawrence *et al*.[46]. The number of publications per cancer genes was determined using PubMed.

### Mapping Cancer Related Missense Mutations on Protein Structures

Somatic missense mutations were mined from the whole genomes in Catalogue of Somatic Mutations in Cancer (COSMIC)[23] and the International Cancer Genome Consortium (ICGC)[22]. The amino acid changes caused by mutations were available in both datasets. We considered only mutations mapped onto canonical proteins in COSMIC and mapped ICGC mutations onto canonical proteins via SNPEff [52]. In total, there were 1,297,414 missense mutations and 543,146 silent mutations in 17,028 tumor samples. We removed any samples from COSMIC that were already present in ICGC to ensure that no mutations were double counted.

### Bi-partite Protein-Residue Networks

Due to the inconsistencies in amino acid sequences between PDB and Uniprot databases, mapping can sometimes fail. When failed mapping resulted in data loss at protein-protein interaction sites (fewer than 5 residues could be mapped for either interacting chain), we discarded the interaction. After mapping PDB to Uniprot residues our network included 2,864 proteins and 3,072 unique PPIs, 199 of which were between cancer genes and their interactors (homo-dimers included).

To determine residues involved in homo-oligomerization, we automatically extracted the self-interacting regions from PDB. If there was a contact region between two chains of the same protein, we assumed that the protein was capable of homo-dimerizing. Since the crystallization process could result in non-biological interactions, we focused on those proteins for which there was evidence in the literature and PDB structures that were predicted to be biological by PISA[43].

### Binding Affinity Calculations

To estimate the change in binding affinity due to a mutation at an interaction interface, we used FoldX[53] version 3.0. As recommended in the FoldX tool’s manual, we discarded the free energy changes smaller than 0.5. Mutations that led to a binding energy change greater than zero were interpreted destabilizing, whereas the ones with binding energy change smaller than zero were interpreted as stabilizing mutations. In cases where there were multiple PDB structures per interaction, we used the consensus of the binding energies per interaction (S3 File). In total, we annotated 1,225 mutations as destabilizing/stabilizing (S2 File).

We used the Reactome Pathway Database[30] to label PPIs with a functional role, namely activating or inhibitory. There were 167 mutations for which the stabilizing/destabilizing effect was predicted and the functional role of the interaction could be assigned a label of activating/inhibitory.

### Mutation Functionality Predictions

We used VEST[29] to classify mutations according to their predicted consequence for protein activity. VEST uses a Random Forest classifier composed of multiple decision trees. VEST scores represent the fraction of decision trees that predicted the mutation to be functional. Thus scores range from 0–1, with 0 representing a unanimous prediction of neutrality, a score of 1 representing a unanimous prediction of a functional mutation, and a score of 0.5 representing an inability to confidently assign either label. We therefore designated mutations receiving a VEST score of 0.75 or higher as functional and mutations with a score of 0.25 or lower as neutral.

### Survival Curves

Clinical data for the 353 patients with TP53 hotspot mutations was downloaded from TCGA. Death due to old age or due to surgical complications can confound survival analysis, therefore we removed individuals that died within the first 30 days after surgery and individuals older than 75 years. We also removed patients that had glioblastoma multiforme or thyroid carcinoma as these diseases have very short and very long survival times respectively relative to most other solid tumors. After filtering, we retained 106 patients with a mutation at residue 273, 68 patients with a mutation at residue 175 and 73 patients with a mutation at residue 248.

We compared a univariate Cox proportional hazards model [54] regressing 5 year survival on primary tumor type to a multivariate model including both tumor type and TP53 hotspot mutations in order to determine whether differences in primary tumor type alone could account for apparent survival differences. This analysis found that the model including TP53 hotspot mutations better explained observed survival differences among cancer patients (P-value < 3.7×10^−2^, ANOVA Test) than a model with tumor type alone. Analyses were performed using the survival package[55] in R[56].

### Ranking Protein Interactions

Interactions were ranked on the basis of the ratio of missense mutations to silent mutations, divided by the ratio of probabilities of the occurrence of a missense versus silent mutation given the codon composition of the interface, similar to the approach in [57]. To determine the probability of a missense mutation resulting from a random nucleotide substitution at an interface, we counted the number of nucleotide sites at the interface that could results in a missense versus a silent mutation. The number of missense versus silent substitutions was pre-calculated for all codons corresponding to each amino acid. Since the exact codon encoding an amino acid cannot be determined from a PDB file, we used the codon with the highest missense substitution count for the given amino acid, as this would give the most conservative estimate of positive selection. A ratio > 1 was considered to be evidence of positive selection for missense mutations at an interface.

## Supporting Information

S1 Fig Summary statistics for genes and mutations used in our analysis. **a)** Violin plots showing the distribution of the number of PDB structures per protein for oncogenes (n = 56), tumor suppressors (n = 47) and other genes (n = 4600) in our analysis. **b)** Violin plots showing the distribution of the number of publications in PubMed associated per protein for oncogenes, tumor suppressors and other genes. **c)** A summary of PDB structures available for cancer genes. “Complex structure” means that there exists at least one structure with a cancer gene bound to another protein or nucleic acid. “Protein structure” means that there is at least a tertiary structure present in the PDB. “Driver genes” simply reflects the number of genes classified as an oncogene or tumor suppressor. **d)** Violin plots showing the distribution of the number of tumor samples with mutations at a given core residue. **e)** Violin plots showing the distribution of the number of tumor sample with mutations at a given interface residue. In **d)** and **e)**, the most frequently altered residues for each set of genes are labeled. **f)** A density plot showing the distribution of VEST scores for mutations occurring at protein core (green), surface non-interface (blue) and interface residues (pink).
(EPS)

S2 Fig Characterizing the structural location of silent mutations in tumor suppressors (TS), oncogenes (OG) and other genes. Fisher’s exact tests were performed separately for each set of genes. Shown are the odds ratios and 95% confidence intervals within each set of genes when making comparing the number of mutations located at **a)** surface versus core residues, **b)** surface interface versus surface non-interface residues.
(EPS)

S1 File Binding affinity calculations per PPI. (XLSX)

S2 File PPI Rankings. (XLSX)

S3 File Binding affinity calculations per PDB and SNP pair. (XLSX)

S1 Table Two-sided Fisher’s exact tests performed to determine structural site-specific enrichment for somatic missense mutations. (DOCX)

S2 Table Two-sided Fisher’s exact test performed to determine enrichment for functional mutations at protein interaction interface residues. (DOCX)

S3 Table The impact of mutations on cancer interactions. Cancer genes and their interaction partners are listed using Entrez gene ids in columns 2 and 4.
(DOCX)

S4 Table Two-sided Fisher’s exact tests performed to determine enrichment for destabilizing mutations in interactions that activate cancer genes. (DOCX)

S5 Table The list of PDB interfaces that were predicted to be biological via PISA webserver. (DOCX)

S6 Table Literature evidence for the homo-oligomerization of driver genes. (DOCX)

S7 Table Two-sided Fisher’s exact tests performed to determine enrichment for functional mutations at homo-oligomerization site residues of cancer genes. (DOCX)

S8 Table The list of 180 proteins that have at least one PDB structure co-complexed with DNA. (DOCX)

S9 Table The list of 141 proteins that have nsSNVs in their DNA binding region. (DOCX)

S10 Table Two-sided Fisher’s Exact Tests Performed to determine enrichment for DNA binding site characteristics. (DOCX)

S11 Table The list of 73 proteins that have at least one PDB structure co-complexed with RNA. (DOCX)

S12 Table The list of 56 proteins that have nsSNVs in their RNA binding region. (DOCX)

S13 Table Two-sided Fisher’s Exact Tests Performed to determine enrichment for RNA binding site characteristics. (DOCX)

S14 Table Two-sided Fisher’s exact tests to determine enrichment of mutations on interactions involving cancer genes. These tests are performed with residue numbers in binding sites or interfaces.
(DOCX)

S15 Table Direct interaction partners of cancer genes that have recently been implicated as cancer genes. (DOCX)

S16 Table 282 Novel cancer genes. (DOCX)
