# Supplementary material for: Structure-Based Analysis Reveals Cancer Missense Mutations Target Protein Interaction Interfaces
Source: PLoS One. 2016 Apr 4;11(4):e0152929. doi: 10.1371/journal.pone.0152929 (PMC4820104; doi:10.1371/journal.pone.0152929)
Supplement: S1 Table — (DOCX) [file pone.0152929.s006.docx]

**S1 Table. Two-sided Fisher’s exact tests performed to determine structural site-specific enrichment for somatic missense mutations**

| **Hypothesis Test** | | **Contingency Table** | | | **P-value** | **Odds Ratio** |
| --- | --- | --- | --- | --- | --- | --- |
| H0: Missense Mutations affect core and surface residues equally.  H1: Missense Mutations are over or under-represented at surface versus core residues. | Tumor Suppressors |  | Surface | Core | 3.6E-02 | 0.84 |
|  |  | Mutated | 1832 | 212 |  |  |
|  |  | Non-mutated | 9872 | 963 |  |  |
|  | Oncogenes |  | Surface | Core | 1.3E-03 | 1.30 |
|  |  | Mutated | 2005 | 179 |  |  |
|  |  | Non-mutated | 10914 | 1268 |  |  |
|  | Other Genes |  | Surface | Core | <2.2E-16 | 1.18 |
|  |  | Mutated | 79391 | 7706 |  |  |
|  |  | Non-mutated | 639636 | 73588 |  |  |
| H0: Silent Mutations affect core and surface residues equally.  H1: Silent Mutations are over or under-represented at surface versus core residues. | Tumor Suppressors |  | Surface | Core | 9.5E-02 | 0.82 |
|  |  | Mutated | 694 | 84 |  |  |
|  |  | Non-mutated | 11010 | 1091 |  |  |
|  | Oncogenes |  | Surface | Core | 9.27E-01 | 0.99 |
|  |  | Mutated | 869 | 99 |  |  |
|  |  | Non-mutated | 12050 | 1348 |  |  |
|  | Other Genes |  | Surface | Core | <2.2E-16 | 0.83 |
|  |  | Mutated | 32587 | 4376 |  |  |
|  |  | Non-mutated | 686440 | 76918 |  |  |
| H0: Missense Mutations are randomly distributed across the surface of proteins.  H1: Missense Mutations are over or under-represented at interface versus surface non-interface residues. | Tumor Suppressors |  | Interface | Surface Non-Interface | 1.4E-04 | 1.28 |
|  |  | Mutated | 381 | 1496 |  |  |
|  |  | Non-mutated | 1666 | 8362 |  |  |
|  | Oncogenes |  | Interface | Surface Non-Interface | 7.92E-03 | 1.17 |
|  |  | Mutated | 402 | 1654 |  |  |
|  |  | Non-mutated | 1908 | 9252 |  |  |
|  | Other Genes |  | Interface | Surface Non-Interface | 4.71E-09 | 0.93 |
|  |  | Mutated | 8994 | 71191 |  |  |
|  |  | Non-mutated | 77176 | 570206 |  |  |

| **Hypothesis Test** | | **Contingency Table** | | | **P-value** | **Odds Ratio** |
| --- | --- | --- | --- | --- | --- | --- |
| H0: Silent Mutations are randomly distributed across the surface of proteins.  H1: Silent Mutations are over or under-represented at interface versus surface non-interface residues. | Tumor Suppressors |  | Interface | Surface Non-Interface | 1.57E-01 | 1.17 |
|  |  | Mutated | 99 | 606 |  |  |
|  |  | Non-mutated | 1364 | 9773 |  |  |
|  | Oncogenes |  | Interface | Surface Non-Interface | 7.58E-01 | 0.95 |
|  |  | Mutated | 74 | 804 |  |  |
|  |  | Non-mutated | 1073 | 11107 |  |  |
|  | Other Genes |  | Interface | Surface Non-Interface | 7.51E-01 | 1.01 |
|  |  | Mutated | 1739 | 31045 |  |  |
|  |  | Non-mutated | 36328 | 653678 |  |  |
