# Supplementary material for: Structure-Based Analysis Reveals Cancer Missense Mutations Target Protein Interaction Interfaces
Source: PLoS One. 2016 Apr 4;11(4):e0152929. doi: 10.1371/journal.pone.0152929 (PMC4820104; doi:10.1371/journal.pone.0152929)
Supplement: S2 Table — (DOCX) [file pone.0152929.s007.docx]

**S2 Table. Two-sided Fisher’s exact test performed to determine enrichment for functional mutations at protein interaction interface residues**

| **Hypothesis Test** | **Contingency Table** | | | **P-value** | **Odds Ratio** |
| --- | --- | --- | --- | --- | --- |
| H0: Functional mutations affect interface and surface non-interface residues equally.  H1: Functional residues are enriched at interface or surface non-interface residues. |  | Interface Residues | Surface Non-Interface Residues | 2.6E-02 | 1.06 |
|  | Functional Mutation | 1885 | 10908 |  |  |
|  | Other Mutations | 4597 | 24823 |  |  |
