# Supplementary material for: Structure-Based Analysis Reveals Cancer Missense Mutations Target Protein Interaction Interfaces
Source: PLoS One. 2016 Apr 4;11(4):e0152929. doi: 10.1371/journal.pone.0152929 (PMC4820104; doi:10.1371/journal.pone.0152929)
Supplement: S3 Table — Cancer genes and their interaction partners are listed using Entrez gene ids in columns 2 and 4. (DOCX) [file pone.0152929.s008.docx]

**S3 Table. The impact of mutations on cancer interactions.** Cancer genes and their interaction partners are listed using Entrez gene ids in columns 2 and 4.

| **Classification of Gene 1** | **Gene 1** | **Mutation on Gene 1** | **Gene 2** | **Binding Energy Change** | **Functionality** |
| --- | --- | --- | --- | --- | --- |
| TS | 1029 | G89V | 1021 | destabilizing | activation |
| OG | 1499 | C429Y | 56998 | destabilizing | inhibition |
| TS | 7428 | R107G | 3091 | destabilizing | activation |
| OG | 2263 | R251L | 2246 | destabilizing | activation |
| OG | 2263 | R251L | 2247 | destabilizing | activation |
| TS | 1029 | T18M | 1021 | stabilizing | activation |
| OG | 2263 | D283N | 2246 | stabilizing | activation |
| OG | 3265 | Q25L | 5894 | stabilizing | activation |
| OG | 1499 | R661L | 56998 | destabilizing | inhibition |
| TS | 7428 | H115Q | 3091 | destabilizing | activation |
| TS | 1029 | G89S | 1021 | destabilizing | activation |
| OG | 1499 | L31W | 8945 | stabilizing | activation |
| TS | 1029 | R87W | 1021 | stabilizing | activation |
| TS | 7428 | W88S | 3091 | destabilizing | activation |
| OG | 1499 | R515Q | 56998 | stabilizing | inhibition |
| TS | 1029 | Y44S | 1021 | destabilizing | activation |
| TS | 1029 | D74V | 1021 | destabilizing | activation |
| TS | 1029 | R24Q | 1021 | stabilizing | activation |
| OG | 2263 | D247N | 2246 | destabilizing | activation |
| OG | 2263 | P253R | 2246 | destabilizing | activation |
| TS | 7428 | R107P | 3091 | destabilizing | activation |
| TS | 324 | A2046T | 8312 | destabilizing | inhibition |
| TS | 4089 | H382D | 4088 | destabilizing | activation |
| TS | 4089 | H382D | 4087 | destabilizing | activation |
| TS | 1029 | N42T | 1021 | destabilizing | activation |
| TS | 7428 | T105P | 3091 | destabilizing | activation |
| TS | 4089 | E337K | 4088 | destabilizing | activation |
| TS | 4089 | E337K | 4087 | stabilizing | activation |
| TS | 1029 | D74E | 1021 | destabilizing | activation |
| TS | 4089 | Y353C | 4088 | destabilizing | activation |
| TS | 4089 | Y353N | 4087 | destabilizing | activation |
| OG | 1499 | I35N | 8945 | destabilizing | activation |
| TS | 4089 | Y353C | 4087 | destabilizing | activation |
| OG | 4233 | F192L | 3082 | destabilizing | activation |
| OG | 2263 | S252W | 2255 | stabilizing | activation |
| TS | 4089 | K507E | 4087 | destabilizing | activation |
| TS | 7157 | K381N | 890 | destabilizing | activation |
| OG | 1499 | I35S | 8945 | destabilizing | activation |
| TS | 1029 | D74N | 1021 | destabilizing | activation |
| TS | 7428 | H110P | 3091 | destabilizing | activation |
| OG | 1499 | H36P | 8945 | destabilizing | activation |
| TS | 7428 | I109S | 3091 | destabilizing | activation |
| OG | 2263 | R251Q | 2253 | destabilizing | activation |
| OG | 2263 | R251Q | 2255 | destabilizing | activation |
| TS | 1029 | T77S | 1021 | destabilizing | activation |
| TS | 7157 | A364T | 7874 | destabilizing | activation |
| OG | 2263 | V280I | 2246 | destabilizing | activation |
| TS | 4089 | H382L | 4088 | stabilizing | activation |
| TS | 7428 | T105M | 3091 | stabilizing | activation |
| TS | 1029 | V51D | 1021 | destabilizing | activation |
| TS | 1029 | R24P | 1021 | stabilizing | activation |
| OG | 2263 | P253R | 2247 | stabilizing | activation |
| TS | 7428 | N67S | 3091 | destabilizing | activation |
| TS | 4089 | V506A | 4087 | destabilizing | activation |
| OG | 1499 | T653R | 56998 | destabilizing | inhibition |
| TS | 1029 | N42Y | 1021 | destabilizing | activation |
| TS | 5925 | F839L | 7027 | destabilizing | activation |
| OG | 1499 | H36R | 8945 | destabilizing | activation |
| OG | 1499 | H36Y | 8945 | stabilizing | activation |
| TS | 5925 | E533G | 1870 | destabilizing | activation |
| OG | 1499 | L31P | 8945 | stabilizing | activation |
| TS | 4089 | V506M | 4088 | destabilizing | activation |
| TS | 4089 | V506M | 4087 | destabilizing | activation |
| OG | 4233 | E221G | 3082 | destabilizing | activation |
| OG | 2263 | P253L | 2247 | stabilizing | activation |
| TS | 1029 | D84N | 1021 | destabilizing | activation |
| OG | 2263 | D247H | 2253 | stabilizing | activation |
| OG | 1499 | R582Q | 56998 | destabilizing | inhibition |
| TS | 4089 | Y353H | 4088 | destabilizing | activation |
| TS | 7428 | H115P | 3091 | destabilizing | activation |
| OG | 2263 | S252W | 2247 | stabilizing | activation |
| TS | 7428 | W88R | 3091 | destabilizing | activation |
| TS | 5925 | E533K | 1869 | destabilizing | activation |
| TS | 7428 | I109N | 3091 | destabilizing | activation |
| OG | 1956 | E114K | 1950 | stabilizing | activation |
| TS | 1029 | V51F | 1021 | destabilizing | activation |
| TS | 7157 | P359S | 7874 | destabilizing | activation |
| TS | 7428 | F91L | 3091 | destabilizing | activation |
| TS | 4089 | Y353N | 4088 | destabilizing | activation |
| OG | 2263 | P253S | 2247 | destabilizing | activation |
| OG | 2263 | P253S | 2246 | destabilizing | activation |
| OG | 1499 | I35M | 8945 | stabilizing | activation |
| TS | 1029 | N42D | 1021 | destabilizing | activation |
| TS | 7428 | W88C | 3091 | destabilizing | activation |
| TS | 4089 | S368C | 4088 | destabilizing | activation |
| TS | 4089 | P356L | 4088 | destabilizing | activation |
| TS | 4089 | P356L | 4087 | destabilizing | activation |
| TS | 7428 | G106D | 3091 | destabilizing | activation |
| TS | 324 | M2047V | 8312 | destabilizing | inhibition |
| TS | 7157 | L383F | 890 | destabilizing | activation |
| OG | 2261 | E322K | 2246 | destabilizing | activation |
| TS | 4089 | P356S | 4087 | destabilizing | activation |
| TS | 4089 | P514S | 4088 | destabilizing | activation |
| TS | 4089 | K507R | 4087 | stabilizing | activation |
| OG | 2263 | R251Q | 2247 | destabilizing | activation |
| OG | 2263 | R251Q | 2246 | destabilizing | activation |
| OG | 1499 | G34E | 8945 | destabilizing | activation |
| TS | 999 | G761E | 1500 | stabilizing | activation |
| TS | 7428 | W88G | 3091 | destabilizing | activation |
| OG | 1956 | D46N | 1950 | stabilizing | activation |
| TS | 4089 | P514S | 4087 | destabilizing | activation |
| TS | 1029 | S56N | 1021 | destabilizing | activation |
| TS | 1029 | A76V | 1021 | stabilizing | activation |
| TS | 7428 | H115Y | 3091 | destabilizing | activation |
| TS | 7428 | G106A | 3091 | destabilizing | activation |
| TS | 7428 | C77S | 3091 | destabilizing | activation |
| OG | 2263 | P253R | 2255 | stabilizing | activation |
| TS | 5925 | E533G | 1869 | destabilizing | activation |
| TS | 1029 | R22P | 1021 | destabilizing | activation |
| TS | 4089 | D355A | 4087 | stabilizing | activation |
| TS | 4089 | D355N | 4087 | stabilizing | activation |
| TS | 4851 | R1937H | 3516 | destabilizing | inhibition |
| OG | 2263 | P253L | 2255 | stabilizing | activation |
| OG | 6502 | R154Q | 6500 | destabilizing | activation |
| TS | 1029 | D84H | 1021 | destabilizing | activation |
| OG | 1499 | I35T | 8945 | destabilizing | activation |
| TS | 1029 | W110C | 1021 | destabilizing | activation |
| OG | 1956 | S492R | 1950 | destabilizing | activation |
| OG | 2263 | D247H | 2247 | stabilizing | activation |
| OG | 2263 | D247H | 2246 | destabilizing | activation |
| TS | 4089 | P356R | 4087 | destabilizing | activation |
| OG | 1956 | Y125C | 7039 | destabilizing | activation |
| TS | 4089 | P356R | 4088 | destabilizing | activation |
| TS | 1029 | R58Q | 1021 | destabilizing | activation |
| OG | 1499 | D32Y | 8945 | destabilizing | activation |
| TS | 1029 | D74A | 1021 | destabilizing | activation |
| TS | 4089 | P356S | 4088 | destabilizing | activation |
| OG | 1956 | Q432H | 7039 | destabilizing | activation |
| OG | 4233 | T230M | 3082 | stabilizing | activation |
| TS | 4089 | V506E | 4087 | destabilizing | activation |
| TS | 4089 | V506E | 4088 | destabilizing | activation |
| OG | 6502 | S291R | 1163 | stabilizing | activation |
| OG | 1499 | G34R | 8945 | destabilizing | activation |
| TS | 4089 | S357P | 4087 | destabilizing | activation |
| OG | 3815 | R205S | 4254 | stabilizing | activation |
| OG | 867 | S80N | 10253 | stabilizing | inhibition |
| TS | 4851 | V2038E | 3516 | destabilizing | inhibition |
| OG | 2263 | A315T | 2247 | destabilizing | activation |
| TS | 7428 | H115D | 3091 | destabilizing | activation |
| TS | 1029 | M53I | 1021 | destabilizing | activation |
| OG | 2263 | D247N | 2253 | stabilizing | activation |
| TS | 7428 | R79G | 3091 | destabilizing | activation |
| OG | 4233 | L229F | 3082 | stabilizing | activation |
| TS | 1029 | M53T | 1021 | destabilizing | activation |
| TS | 7428 | W88L | 3091 | destabilizing | activation |
| TS | 4089 | E337G | 4088 | destabilizing | activation |
| TS | 7428 | H110N | 3091 | destabilizing | activation |
| TS | 5925 | E533K | 1870 | destabilizing | activation |
| OG | 1499 | D390E | 56998 | stabilizing | inhibition |
| TS | 4851 | V2038L | 3516 | destabilizing | inhibition |
| TS | 5295 | H669Q | 5159 | destabilizing | activation |
| OG | 1499 | R469H | 56998 | destabilizing | inhibition |
| OG | 2263 | P253S | 2255 | destabilizing | activation |
| TS | 1029 | D84Y | 1021 | destabilizing | activation |
| TS | 4089 | R380K | 4088 | stabilizing | activation |
| OG | 1499 | C619Y | 56998 | destabilizing | inhibition |
| TS | 7428 | Y112D | 3091 | destabilizing | activation |
| TS | 4089 | R380K | 4087 | stabilizing | activation |
| OG | 1499 | G34I | 8945 | destabilizing | activation |
| TS | 4089 | D355G | 4088 | destabilizing | activation |
| TS | 4089 | D355G | 4087 | stabilizing | activation |
| TS | 4089 | D355V | 4087 | stabilizing | activation |
| OG | 1499 | G34V | 8945 | destabilizing | activation |
| TS | 5925 | R857H | 1869 | destabilizing | activation |
| OG | 1499 | N387Y | 56998 | destabilizing | inhibition |
| TS | 1029 | D74Y | 1021 | destabilizing | activation |
| OG | 2263 | R165W | 2246 | stabilizing | activation |
