# Supplementary material for: Structure-Based Analysis Reveals Cancer Missense Mutations Target Protein Interaction Interfaces
Source: PLoS One. 2016 Apr 4;11(4):e0152929. doi: 10.1371/journal.pone.0152929 (PMC4820104; doi:10.1371/journal.pone.0152929)
Supplement: S4 Table — (DOCX) [file pone.0152929.s009.docx]

**S4 Table. Two-sided Fisher’s exact tests performed to determine enrichment for destabilizing mutations in interactions that activate cancer genes**

| **Hypothesis Test** | **Contingency Table** | | | **P-value** | **Odds Ratio** |
| --- | --- | --- | --- | --- | --- |
| H0: Destabilizing mutations affect activating interactions of TSs and OGs equally.   H1: Destabilizing mutations are over or underrepresented at activating interactions of TSs versus OGs. |  | TS | OG | 8.36E-04 | 3.65 |
|  | Destabilizing | 83 | 31 |  |  |
|  | Stabilizing | 16 | 22 |  |  |
