# Supplementary material for: Structure-Based Analysis Reveals Cancer Missense Mutations Target Protein Interaction Interfaces
Source: PLoS One. 2016 Apr 4;11(4):e0152929. doi: 10.1371/journal.pone.0152929 (PMC4820104; doi:10.1371/journal.pone.0152929)
Supplement: S5 Table — (DOCX) [file pone.0152929.s010.docx]

**S5 Table. The list of PDB interfaces that were predicted to be biological via PISA webserver.**

| **HomoDimer** | **Biological PDB interfaces** |
| --- | --- |
| ABL1 | 2g2iAB, 3qriAB, 3qrjAB, 2o88AB, 3eg1AB, 2g1tAB |
| APC | 1debAB |
| B2M | 3myzAB, 3ciqAB, 3lowAB, 4e0lAB, 4e0kAB, 3tm6AB |
| BCL2 | 2xa0AB |
| BRAF | 4ehgAB, 1uwhAB, 3prfAB, 3tv4AB, 3ii5AB, 3skcAB, 3ny5AB, 4eheAB, 4mbjAB |
| CBL | 2oo9AB, 3plfBD |
| EGFR | 2jiuAB, 3ikaAB, 4g5pAB, 3b2uAI, 4i24AB, 2gs7AB, 2jivAB, 2jitAB, 3gt8AD |
| ERBB2 | 4hrnCD, 3h3bAB, 3rcdAB, |
| FGFR2 | 2pwlAB, 3ri1AB, 4j97AB, 2q0bAB, 2pvyAC, 4j98AB, 4j99AB, 3b2tAB, 4j95AB, 2py3AB,2psqAB, 2pz5AB, 2pzrAB , 2pzpAB, 4j96AB |
| HNF1A | 2gypAB, 1ic8AB |
| HRAS | 3lo5AC |
| IDH1 | 3inmAB, 4kzoAB, 4l06AB, 3mapAB, 1t0lAB, 4i3kAB, 4l04AB, 4i3lAB, 3marAB, 3masAB, 4l03AB |
| KIT | 1pkgAB, 3g0fAB |
| MAGI1 | 2r4hAB |
| MDM2 | 3vbgAB, 3tpxAC, 4hbmAB, 3lnjAC, 3w69AB, 3v3bAB, 3tj2AC, 3iwyAC, 4dijAB |
| MET | 3efjAB, 2uzxBD, 3efkAB, 2rfnAB |
| NF1 | 3p7zAB |
| NOTCH1 | 3etoAB, 3i08AC |
| PPP2R1A | 1b3uAB |
| RET | 2x2mAB, 2ivsAB |
| SMAD4 | 1g88AB, 1dd1AB |
| SPOP | 3htmAB, 3hqiAB, 3hu6AB |
| TNFAIP3 | 3zjeAB, 3zjgAB, 3zjfAB |
| TP53 | 2ac0AB, 3ts8AB, 1tupAB, 4hjeAB, 3q06AB, 3d0aAB, 3q01AB, 1tsrAB |
