# Supplementary material for: Structure-Based Analysis Reveals Cancer Missense Mutations Target Protein Interaction Interfaces
Source: PLoS One. 2016 Apr 4;11(4):e0152929. doi: 10.1371/journal.pone.0152929 (PMC4820104; doi:10.1371/journal.pone.0152929)
Supplement: S6 Table — (DOCX) [file pone.0152929.s011.docx]

**S6 Table. Literature evidence for the homo-oligomerization of driver genes.**

| **Gene** | **PMID** | **Gene** | **PMID** |
| --- | --- | --- | --- |
| ABL1 | 12591740 | IDH2 | 23680144 |
| APC | 11099951 | JAK2 | 24218541 |
| B2M | 20088607 | KIT | 12879016 |
| BCL2 | 7937747 | KRAS | 24803535 |
| BRAF | 16858395 | MAGI1 | 17267502 |
| BRCA1 | 10026184 | MDM2 | 18219319 |
| CASP8 | 23773332 | MET | 15261142 |
| CBL | 12842890 | NF1 | 9278498 |
| CREBBP | 11572963 | NF2 | 22750751 |
| DNMT1 | 19173286 | NOTCH1 | 15561108 |
| EGFR | 11597398 | PPP2R1A | 25659743 |
| ERBB2 | 16707444 | RET | 9879991 |
| FGFR2 | 22787153 | RUNX1 | 17355962 |
| GATA3 | 18621058 | SMAD4 | 21724602 |
| HNF1A | 22445113 | SPOP | 19818708 |
| HRAS | 24516166 | TNFAIP3 | 23602765 |
| IDH1 | 19435942 | TP53 | 1554407 |
