# Supplementary material for: Structure-Based Analysis Reveals Cancer Missense Mutations Target Protein Interaction Interfaces
Source: PLoS One. 2016 Apr 4;11(4):e0152929. doi: 10.1371/journal.pone.0152929 (PMC4820104; doi:10.1371/journal.pone.0152929)
Supplement: S7 Table — (DOCX) [file pone.0152929.s012.docx]

**S7 Table. Two-sided Fisher’s exact tests performed to determine enrichment for functional mutations at homo-oligomerization site residues of cancer genes**

| **Hypothesis Test** | **Contingency Table** | | | **P-value** | **Odds Ratio** |
| --- | --- | --- | --- | --- | --- |
| H0: Functional mutations are equally prevalent at homo-oligomerization sitesof tumor suppressorss.   H1: Functional mutations are over or underrepresented on the homo-oligomerization sites of tumor suppressors. |  | Functional Mutations | Other Mutations | 1.73E-08 | 3.68 |
|  | Residues involved in homo-oligomerization of  tumor suppressors | 37 | 47 |  |  |
|  | All Residues of  Other Genes | 20092 | 94063 |  |  |
| H0: Functional mutations are equally prevalent at homo-oligomerization sitesof oncogenes.   H1: Functional mutations are over or underrepresented on the homo-oligomerization sites of oncogenes. |  | Functional Mutations | Other Mutations | 3.01E-01 | 0.77 |
|  | Residues involved in homo-oligomerization of  oncogenes | 23 | 139 |  |  |
|  | All Residues of  Other Genes | 20092 | 94063 |  |  |
| H0: Functional mutations are equally prevalent at homo-oligomerization sitesof cancer genes.   H1: Functional mutations are over or underrepresented on the homo-oligomerization sites of cancer genes. |  | Functional Mutations | Other Mutations | 6.40E-07 | 0.21 |
|  | Residues involved in homo-oligomerization of  Tumor Suppressors | 37 | 47 |  |  |
|  | Residues involved in homo-oligomerization of  Oncogenes | 23 | 139 |  |  |
