# Supplementary material for: Structure-Based Analysis Reveals Cancer Missense Mutations Target Protein Interaction Interfaces
Source: PLoS One. 2016 Apr 4;11(4):e0152929. doi: 10.1371/journal.pone.0152929 (PMC4820104; doi:10.1371/journal.pone.0152929)
Supplement: S8 Table — (DOCX) [file pone.0152929.s013.docx]

**S8 Table.** **The list of 180 proteins that have at least one PDB structure co-complexed with DNA.**

| ADAR | ESR1 | HIST1H2BA | MBD1 | NR5A2 | RFC1 | TFAM |
| --- | --- | --- | --- | --- | --- | --- |
| AGER | ESRRB | HIST1H2BJ | MBD4 | OGG1 | RFX1 | TFDP2 |
| AIM2 | ETS1 | HIST1H2BK | MECP2 | PARP1 | RNASEH1 | THAP1 |
| ALKBH2 | ETS2 | HIST1H3F | MEF2A | PAX3 | RPA1 | THRB |
| APEX1 | ETV1 | HIST2H3D | MEF2BNB | PAX6 | RUNX1 | TOP1 |
| APTX | EXO1 | HIST2H4A | MGMT | PBX1 | RXRA | TOP2B |
| ARID5B | F2 | HIST3H3 | MPG | PCBP1 | SATB1 | TP53 |
| ARNTL | FEN1 | HMBOX1 | MSH2 | PCBP2 | SMAD3 | TP63 |
| ATF2 | FOS | HMGA1 | MSH3 | PDGFB | SOX2 | TP73 |
| BANF1 | FOXA3 | HNF1A | MSH6 | PGR | SOX9 | U2AF2 |
| BLM | FOXK2 | HNF1B | MSL3 | PITX2 | SPDEF | UHRF1 |
| CEBPB | FOXM1 | HNF4A | MTERF1 | POLB | SREBF1 | UHRF2 |
| CENPA | FOXO1 | HNRNPA1 | MXD1 | POLH | SRF | UNG |
| CENPB | FOXO3 | HNRNPD | MYC | POLI | SRY | USF1 |
| CLOCK | FOXO4 | HNRNPK | NABP2 | POLK | STAT1 | VDR |
| CNOT6L | FOXP2 | HOXB1 | NFAT5 | POLL | SUB1 | VWF |
| CXXC1 | FOXP3 | IFI16 | NFATC1 | POLM | TAL1 | WRN |
| DDB2 | FUBP1 | IL6 | NFATC2 | POT1 | TARDBP | WT1 |
| DNMT1 | GATA3 | IRF3 | NFKB1 | POU2AF1 | TBP | XRCC5 |
| DR1 | GBX1 | JUN | NFKB2 | POU2F1 | TBX1 | XRCC6 |
| DRAP1 | GLI1 | KMT2A | NFYA | POU6F1 | TCF3 | YY1 |
| E2F4 | GTF2B | KMT2B | NFYB | PUF60 | TDG | ZBP1 |
| ELK1 | H2AFV | LIG1 | NFYC | RARA | TDP1 | ZBTB33 |
| ELK4 | H2AFZ | LIG3 | NKX2-5 | RBPJ | TERF1 | ZNF217 |
| ERCC4 | H3F3B | MAFA | NR1D1 | RECQL | TERF2 |  |
| ERG | HIST1H2AB | MAX | NR1H2 | RELA | TET2 |  |
