# Supplementary material for: Structure-Based Analysis Reveals Cancer Missense Mutations Target Protein Interaction Interfaces
Source: PLoS One. 2016 Apr 4;11(4):e0152929. doi: 10.1371/journal.pone.0152929 (PMC4820104; doi:10.1371/journal.pone.0152929)
Supplement: S9 Table — (DOCX) [file pone.0152929.s014.docx]

**S9 Table.** **The list of 141 proteins that have nsSNVs in their DNA binding region.**

| MYC | RXRA | RECQL | HIST3H3 | HNF4A | PARP1 |
| --- | --- | --- | --- | --- | --- |
| TET2 | H2AFV | SPDEF | MSH3 | CENPB | AIM2 |
| GATA3 | NABP2 | IRF3 | HIST2H3D | TP73 | HNRNPK |
| TP53 | POLI | MAFA | TFAM | POLB | MGMT |
| WT1 | NKX2-5 | HMGA1 | TERF2 | SUB1 | FOXP3 |
| SOX9 | TDG | MPG | TERF1 | MBD4 | SRY |
| RUNX1 | POU6F1 | DDB2 | HOXB1 | POT1 | NFKB1 |
| MSH2 | SATB1 | HIST1H2BK | TOP2B | ESRRB | NFKB2 |
| MSH6 | POLL | ALKBH2 | TOP1 | GLI1 | MBD1 |
| HNF1A | PITX2 | KMT2A | ETS1 | POLK | THRB |
| FOXO4 | SOX2 | NFYA | PAX6 | MXD1 | FEN1 |
| FOXM1 | IL6 | FOXO1 | PGR | SMAD3 | ELK1 |
| TP63 | NR5A2 | NFYC | NR1H2 | GBX1 | TDP1 |
| HIST1H2AB | F2 | XRCC6 | STAT1 | ZBTB33 | ELK4 |
| JUN | NR1D1 | PCBP1 | H2AFZ | KMT2B | LIG3 |
| ESR1 | ZBP1 | PCBP2 | NFATC1 | TBX1 | GTF2B |
| PAX3 | CLOCK | H3F3B | NFATC2 | CEBPB |  |
| MAX | HIST1H2BJ | APEX1 | TARDBP | POU2AF1 |  |
| VWF | RARA | HIST1H3F | FOXA3 | POU2F1 |  |
| RFC1 | YY1 | ERCC4 | U2AF2 | HNRNPD |  |
| ARNTL | POLM | BLM | ETS2 | FOXP2 |  |
| ARID5B | MTERF1 | BANF1 | ETV1 | ZNF217 |  |
| CXXC1 | MEF2BNB | VDR | FOXK2 | E2F4 |  |
| USF1 | MECP2 | HMBOX1 | HNRNPA1 | MEF2A |  |
| NFAT5 | TBP | RELA | WRN | FOXO3 |  |
