# Supplementary material for: Structure-Based Analysis Reveals Cancer Missense Mutations Target Protein Interaction Interfaces
Source: PLoS One. 2016 Apr 4;11(4):e0152929. doi: 10.1371/journal.pone.0152929 (PMC4820104; doi:10.1371/journal.pone.0152929)
Supplement: S10 Table — (DOCX) [file pone.0152929.s015.docx]

**S10 Table.** **Two-sided Fisher’s Exact Tests Performed to determine enrichment for DNA binding site characteristics**

| **Hypothesis Test** | **Contingency Table** | | | **P-value** | **Odds Ratio** |
| --- | --- | --- | --- | --- | --- |
| H0: DNA binding residues participate in protein interaction sites proportional to the number of protein interface residues.  H1: DNA binding residues are over- or underrepresented at protein interfaces. |  | DNA Binding Site | Other Residues | 8.86E-05 | 0.77 |
|  | Interface | 240 | 3690 |  |  |
|  | Non-Interface | 2838 | 33453 |  |  |
| H0: Missense mutations affect all DNA binding protein residues equally.   H1: Missense mutations are over- or underrepresented at DNA binding sites. |  | DNA Binding Site | Other Residues | 3.38E-03 | 1.19 |
|  | Mutated | 374 | 3874 |  |  |
|  | Non-mutated | 2704 | 33269 |  |  |
| H0: Silent mutations affect all DNA binding protein residues equally   H1: Silent mutations are over- or under- represented at DNA binding sites. |  | DNA Binding Site | Other Residues | 8.6E-01 | 0.98 |
|  | Mutated | 142 | 1745 |  |  |
|  | Non-mutated | 2936 | 35398 |  |  |
