# Supplementary material for: Structure-Based Analysis Reveals Cancer Missense Mutations Target Protein Interaction Interfaces
Source: PLoS One. 2016 Apr 4;11(4):e0152929. doi: 10.1371/journal.pone.0152929 (PMC4820104; doi:10.1371/journal.pone.0152929)
Supplement: S11 Table — (DOCX) [file pone.0152929.s016.docx]

**S11 Table.** **The list of 73 proteins that have at least one PDB structure co-complexed with RNA.**

| ADAR | DHX9 | IFIH1 | OAS1 | PUM2 | SLBP | SNRPF | SSB |
| --- | --- | --- | --- | --- | --- | --- | --- |
| AGO1 | DICER1 | IFIT5 | PABPC1 | QKI | SNRPA | SNRPG | SUPV3L1 |
| AGO2 | EIF4A3 | IGHMBP2 | PARN | RBFOX1 | SNRPA1 | SRP14 | TARBP2 |
| CASC3 | ELAVL1 | LIN28A | PCBP2 | RBMX | SNRPB | SRP19 | TRA2B |
| CELF1 | ELAVL4 | NHP2L1 | PHAX | RBMY1A1 | SNRPB2 | SRP54 | U2AF2 |
| CMTR1 | ERI1 | NOVA1 | PIWIL1 | RNASEH1 | SNRPD1 | SRP68 | WARS |
| CPSF6 | FMR1 | NOVA2 | PRPF31 | RNASEL | SNRPD2 | SRP9 | ZFP36L2 |
| DDX19B | GARS | NUDT21 | PTBP1 | SEPSECS | SNRPD3 | SRSF1 | ZNF346 |
| DDX58 | HNRNPF | NXF1 | PUM1 | SF1 | SNRPE | SRSF2 | ZRANB2 |
| DHX58 |  |  |  |  |  |  |  |
