# Supplementary material for: Structure-Based Analysis Reveals Cancer Missense Mutations Target Protein Interaction Interfaces
Source: PLoS One. 2016 Apr 4;11(4):e0152929. doi: 10.1371/journal.pone.0152929 (PMC4820104; doi:10.1371/journal.pone.0152929)
Supplement: S12 Table — (DOCX) [file pone.0152929.s017.docx]

**S12 Table.** **The list of 56 proteins that have nsSNVs in their RNA binding region.**

| SRSF2 | SNRPB2 | WARS | AGO2 | RBFOX1 | SNRPD2 | ELAVL4 |
| --- | --- | --- | --- | --- | --- | --- |
| DDX58 | SNRPA | NHP2L1 | ZRANB2 | CMTR1 | SNRPE | OAS1 |
| CELF1 | PUM2 | PABPC1 | NXF1 | SRSF1 | IFIT5 | RBMX |
| PHAX | SRP19 | PCBP2 | SF1 | IFIH1 | IGHMBP2 | SEPSECS |
| NUDT21 | SRP54 | PTBP1 | SLBP | SRP68 | FMR1 | PUM1 |
| DICER1 | U2AF2 | HNRNPF | PIWIL1 | AGO1 | DHX58 | PRPF31 |
| SSB | DDX19B | ERI1 | QKI | TRA2B | ELAVL1 | NOVA1 |
| SNRPB | LIN28A | RNASEL | ZFP36L2 | ADAR | EIF4A3 | DHX9 |
