# Supplementary material for: Structure-Based Analysis Reveals Cancer Missense Mutations Target Protein Interaction Interfaces
Source: PLoS One. 2016 Apr 4;11(4):e0152929. doi: 10.1371/journal.pone.0152929 (PMC4820104; doi:10.1371/journal.pone.0152929)
Supplement: S13 Table — (DOCX) [file pone.0152929.s018.docx]

**S13 Table.** **Two-sided Fisher’s Exact Tests Performed to determine enrichment for RNA binding site characteristics**

| **Hypothesis Test** | **Contingency Table** | | | **P-value** | **Odds Ratio** |
| --- | --- | --- | --- | --- | --- |
| H0: Missense mutations affect all RNA binding protein residues equally.   H1: Missense mutations are over- or underrepresented at RNA binding sites. |  | RNA Binding Site | Other Residues | 1.27E-02 | 1.23 |
|  | Mutated | 190 | 1565 |  |  |
|  | Non-mutated | 1373 | 13921 |  |  |
| H0: Silent mutations affect all RNA binding protein residues equally.   H1: Silent mutations are over- or under- represented at RNA binding sites. |  | RNA Binding Site | Other Residues | 7.06E-01 | 1.04 |
|  | Mutated | 76 | 724 |  |  |
|  | Non-mutated | 1487 | 14762 |  |  |
