# Supplementary material for: Structure-Based Analysis Reveals Cancer Missense Mutations Target Protein Interaction Interfaces
Source: PLoS One. 2016 Apr 4;11(4):e0152929. doi: 10.1371/journal.pone.0152929 (PMC4820104; doi:10.1371/journal.pone.0152929)
Supplement: S14 Table — These tests are performed with residue numbers in binding sites or interfaces. (DOCX) [file pone.0152929.s019.docx]

**S14 Table. Two-sided Fisher’s exact tests to determine enrichment of mutations on interactions involving cancer genes.** These tests are performed with residue numbers in binding sites or interfaces.

| **Hypothesis Test** | **Contingency Table** | | | **P-value** | **Odds Ratio** |
| --- | --- | --- | --- | --- | --- |
| H0: All edges in the network are equally likely to be mutated.   H1: Mutations are over- or underrepresented at edges involving a cancer gene. |  | Cancer Driver Edges | Other Edges | <2.2E-16 | 1.62 |
|  | Mutated | 999 | 8486 |  |  |
|  | Non-mutated | 5275 | 72742 |  |  |
| H0: Mutations affecting cancer gene interactions are equally distributed on both interaction partners.  H1: Mutations affecting cancer gene interactions are over- or under- represented on cancer genes relative to their binding partners. |  | Cancer Gene Binding Sites | Partner Binding Sites | 4.59E-09 | 1.53 |
|  | Mutated | 771 | 280 |  |  |
|  | Non-mutated | 4440 | 2470 |  |  |
