# Supplementary material for: Structure-Based Analysis Reveals Cancer Missense Mutations Target Protein Interaction Interfaces
Source: PLoS One. 2016 Apr 4;11(4):e0152929. doi: 10.1371/journal.pone.0152929 (PMC4820104; doi:10.1371/journal.pone.0152929)
Supplement: S15 Table — (DOCX) [file pone.0152929.s020.docx]

**S15 Table. Direct interaction partners of cancer genes that have recently been implicated as cancer genes.**

| CBFB |
| --- |
| CD1D |
| CDK4 |
| CDKN1B |
| HLA-A |
| HLA-B |
| KEAP1 |
| MLL |
| RASA1 |
| SOS1 |
| TCEB1 |
| TCF7L2 |
| TP53BP1 |
