# Supplementary material for: Structure-Based Analysis Reveals Cancer Missense Mutations Target Protein Interaction Interfaces
Source: PLoS One. 2016 Apr 4;11(4):e0152929. doi: 10.1371/journal.pone.0152929 (PMC4820104; doi:10.1371/journal.pone.0152929)
Supplement: S16 Table — (DOCX) [file pone.0152929.s021.docx]

**S16 Table. 282 Novel cancer genes.**

| ADAM17 | CD74 | EPHA4 | HLA-DRB1 | JUN | NDNL2 | PRIM1 | S100A4 | TLR2 |
| --- | --- | --- | --- | --- | --- | --- | --- | --- |
| AGXT | CDC16 | ETFA | HLA-DRB5 | KAT6A | NEDD4 | PRIM2 | SCN5A | TMEM189-UBE2V1 |
| ANGPT1 | CDC26 | ETFB | HLA-E | KAT8 | NEDD4L | PRMT5 | SCNN1A | TNFRSF10B |
| APAF1 | CDC37 | EXOSC2 | HLA-G | KDM1B | NFATC2 | PROC | SEC23A | TNFRSF11B |
| APBA1 | CDC42 | EXOSC7 | HNF4A | KLRC1 | NGFR | PROCR | SEC24A | TNFRSF4 |
| APOH | CDK2 | FGA | HOXC9 | KLRD1 | NPPC | PTPN13 | SERPINE1 | TNFSF10 |
| APP | CDK8 | FGB | HSP90AA1 | KLRF2 | NPR3 | PTPRZ1 | SETD1A | TNFSF11 |
| ARHGAP1 | CDT1 | FGFR1 | IDE | XRCC1 | NR3C1 | PVR | SETD1B | TNFSF4 |
| ARHGEF11 | CFB | FMN2 | IFNA2 | KRIT1 | NSMCE1 | RAB4A | SFN | TNK2 |
| ARHGEF25 | CHMP1A | FN1 | IFNAR2 | LCN2 | NTF4 | RAD18 | SH2D1A | TNRC6C |
| ARHGEF7 | CHMP4B | FRS2 | IL13RA1 | LDLR | NTNG2 | RAF1 | SKP1 | TOB1 |
| ARL1 | CLEC2A | FYN | IL1B | LMO2 | NTRK2 | RALB | SLAMF1 | TRIM24 |
| ATXN2 | CLIP1 | GAS6 | IL1R2 | LRP6 | NXF1 | RALBP1 | SNRPD2 | TRIO |
| AURKB | CNOT7 | GDF5 | IL1RAP | LRRC4 | NXT1 | RAMP2 | SNRPF | TUBA1B |
| AXL | CNTN1 | GH1 | IL1RL1 | MAP2K5 | PABPC1 | RAN | SNUPN | UBA3 |
| BAK1 | COMMD3-BMI1 | GHR | IL21 | MAPK1 | PAIP2 | RANBP1 | SOCS2 | UBE2B |
| BCL2A1 | CR2 | GMNN | IL21R | MAPK14 | PAK1 | RAP1B | SPIRE1 | UBE2D2 |
| BMPR1A | CSTA | GNAI1 | IL22 | MAPK7 | PDCD6IP | RAPGEF6 | SRPK1 | UBE2I |
| BRK1 | CTSL | GOLGA4 | IL22RA2 | MAPKAPK2 | PDE6D | RARA | SRSF1 | UBE2M |
| C11orf30 | CUL1 | GSPT1 | IL33 | MAX | PDHA1 | RBSN | SRXN1 | UBE2N |
| C1QA | CYFIP1 | GSTM2 | IL4R | MCM6 | PDHB | RGS16 | SUMO1 | USH1C |
| C1QB | DCUN1D1 | GSTM3 | IL5 | MEF2B | PDK3 | RLN3 | SUMO2 | USH1G |
| C1QC | DKK1 | GUCY1A3 | IL5RA | MICALL1 | PEX19 | RND1 | TAF12 | VTN |
| C3 | DLAT | GUCY1B3 | INCENP | MITD1 | PEX3 | RND3 | TAF4 | WDR5 |
| CABIN1 | DOCK2 | H2AFX | INSL5 | MMP10 | PEX5 | RNF2 | TAL1 | WDR77 |
| CALCRL | EEF1A2 | HEXA | INSR | MORF4L1 | PHF12 | ROCK1 | TCF3 | XIAP |
| CALM1 | EFNA2 | HEXB | IRS1 | MSL1 | PLEC | RPGR | TEK |  |
| CASP9 | EFNA5 | HIST1H2AE | ITGAX | MXD1 | PLXNB1 | RPL4 | TGFB3 |  |
| CBX1 | EFNB2 | HIST1H2BJ | ITGB2 | MYH9 | PNKP | RPL7 | TIGIT |  |
| CCL3 | EHD1 | HIST1H2BK | ITGB3 | NAE1 | POLR2D | RPS3 | TIMP1 |  |
| CCNA2 | EIF4G1 | HIST1H3A | ITGB4 | NCOA1 | POLR2G | RPS6KA1 | TIMP3 |  |
| CCNC | ELMO1 | HLA-DRA | ITSN1 | NCOA2 | PRDX1 | RPS7 | TLR1 |  |
